# Benchmarking within-sample minority variant detection with short-read sequencing in *M. tuberculosis*

**DOI:** 10.64898/2026.02.13.704885

**Published:** 2026-02-16

**Authors:** Shandukani Mulaudzi, Sanjana Kulkarni, Maximillian G. Marin, Maha R Farhat

**Affiliations:** 1 Department of Biomedical Informatics, Harvard University, 10 Shattuck St, Boston, MA 02115, USA; 2 Department of Data Science, Dana-Farber Cancer Institute, 450 Brookline Avenue, Boston, MA 02215, USA; 3 Division of Pulmonary and Critical Care, Department of Medicine, Massachusetts General Hospital, 55 Fruit Street Boston, MA 02114, USA

**Keywords:** Low-frequency variants, variant calling, whole genome sequencing, *Mycobacterium tuberculosis*, short-reads, benchmark, low mappability regions, reference bias

## Abstract

**Background:**

Low-frequency (minority) variants—variants detectable within a sample at low allele frequencies—are relevant in several areas of research and health, ranging from cancer to pathogen heteroresistance. There is uncertainty around the optimal bioinformatic approach to accurately and reproducibly distinguish low-frequency variants from sequencing or mapping error. To address this we benchmarked seven variant callers on precision, recall and false positive characteristics for detecting low-frequency variants using simulated short-read whole genome sequencing data for 700 *Mycobacterium tuberculosis* strains. We developed a new low-frequency error model for filtering output of the best performing tool using read mapping and quality metrics.

**Results:**

We simulated 378 unique variants across 5 genomic backgrounds spanning 4 lineages. Variants were simulated to represent 3 genomic region categories, 10 allele frequencies and 5 sequencing depths. FreeBayes, a haplotype-based variant caller, achieved the highest pooled F1 score of the seven tools in drug resistance regions (average F1 = 0.86) and its higher performance held across genomic context and background. Across tools, we identified lower performance in repetitive (low mappability) regions, and strong reference bias in low-frequency variant calling. We validated variant caller performance on a sample of *in-vitro* strain mixtures substantiating our ranking. When paired with FreeBayes, the error model excludes 49% of false variants and <1% of true variants.

**Conclusions:**

Our analysis provides evidence to support best practices for low-frequency variant calling, including tool choice, masking and filtering. We also develop and provide a new error model that excludes false positive low-frequency variant calls from FreeBayes output.

## Background

Bulk whole-genome sequencing (WGS) is usually applied to study genetic variation that differentiates distinct cell populations. However, bulk WGS can also capture recently evolved diversity emerging within a single population. The latter requires the accurate detection of low-frequency (minority) or sub-consensus variants present at mapped read frequencies typically <75–95%. Low-frequency variants are relevant for understanding within-host pathogen evolution, somatic oncogenesis in cancers, and microbiome diversity, among other applications. Specifically for *Mycobacterium tuberculosis* (*Mtb*), the causative agent of tuberculosis (TB), patient-derived *Mtb* isolates from bacterial culture are not pure clones.^1–5^ Bulk *Mtb* whole-genome sequencing at medium-to-high depth suggests that up to 85% of variant calls per sample occur at mapped read frequencies of less than 50%.^1,6–10^ This variation may have been present at infection (e.g. multi-strain primary infections) or may have arisen during reinfection with additional *Mtb* strain(s). Low-frequency variation can also arise during the course of chronic clonal infection when subpopulations of bacteria acquire new variants in-host.^11–14^ Some of these low-frequency variants have been shown to rise to fixation in-host, and within-population frequency of 19% or more have been shown to predict subsequent fixation in-host.^15–17,14,18,19^ Low-frequency variants therefore present an opportunity for early diagnosis of drug resistance (DR) in *Mtb* or early detection of poor TB treatment response, as has been shown previously for *Mtb* and other pathogens.^20–22^

Although whole-genome sequencing enables the detection of low-frequency variants, such variants are hard to distinguish from base level sequencing error especially at lower depths, or from mapping error due to sequence homology or reference bias. Previous *Mtb* WGS studies have employed several variant calling approaches to distinguish between true and false low-frequency variants including: (1) hard filtering variant calls on quality metrics including depth, allele frequency, base quality, strand bias,^9,23–26,19,27,28^ (2) variant calling against a more closely matched reference genome,^25^ (3) excluding variants in repetitive regions,^1,6,8,24,29^ (4) empiric comparison to data from deep targeted sequencing,^30^ and (5) taking a consensus of variant calls across two or more tools.^6,31^ Despite the empiric application of these different bioinformatic approaches, data guiding the choice of variant calling tool is currently lacking and this significantly limits the interpretation of the results of previous analyses. In this *Mtb*-focused benchmarking analysis we explicitly consider the effects of repetitive sequence and homopolymeric context as well as reference bias on low-frequency variant calling, surpassing existing benchmarking efforts on minority variants in gut microbiome, viral and human tumor data.^32–39^

We assess the performance of seven tools on simulated data, providing one of the most comprehensive benchmarking studies to date for low-frequency within-sample variant calling from bulk sequence data. Tools were selected either because they were specifically developed for *Mtb* low-frequency variant detection (binoSNP)^40^ or general microbial variant detection (Pilon),^41^ are commonly applied for *Mtb* low-frequency variant calling (LoFreq, VarScan2),^42,43^ or because they are highly cited methods for somatic mutation calling in human data (FreeBayes, Mutect2, VarDict).^44–46^ Specifically we benchmark: (1) FreeBayes: a haplotype-based bayesian caller used for SNV and small indel genotyping in the second edition of the WHO catalog of MTBC DR mutations,^47^ (2) LoFreq: an ultra-sensitive variant caller for uncovering cell-population heterogeneity, (3) Mutect2: GATK’s recommended variant caller for somatic short variant discovery, (4) Pilon: an integrated tool for microbial variant calling and genome assembly, (5) VarDict: developed for next-generation sequencing in cancer research, and previously benchmarked against FreeBayes and other variant callers for bacterial WGS data,^48^ (6) VarScan2: a tool for somatic mutation and copy number alteration discovery in cancer, and (7) BinoSNP: developed for low-frequency detection of single nucleotide variants (SNVs) in *Mtb* complex (MTBC) strains. We simulate WGS data using two different approaches (InSilicoSeq and ART).^49,50^ Each simulated *Mtb* strain has a distinct combination of 1) mutant background 2) simulated depth, 3) mutant allele frequency and 4) reference genome. The simulations are replicated five times for a total of 7,000 WGS simulations.

## Results

### Simulations

We benchmarked seven variant callers (Methods) for low-frequency variant detection in *Mtb*. We simulated 500 different mutant H37Rv strains in duplicate using InSilicoSeq (ISS) and ART. Each strain had a distinct combination of 10 different haplotypes of 50 mutations spanning drug resistance–DR, low mappability–LM, or homopolymeric tract–HT regions (Methods), simulated depths (5 depths ranging 50 to 700x), and mutant allele frequencies (AFs, 10 AFs ranging 1% to 50%). Strains were simulated in five random replicates for a total of 5,000 WGS simulations. In a second set of simulations, 50 mutant strains were simulated from four non-H37Rv reference genomes belonging to L1, L2, L3 and non-H37Rv L4 respectively. Sequencing depth and variant allele frequency were varied as above for 20 DR SNVs per strain. Both ISS and ART were used to simulate five replicates for a total of 2,000 WGS simulations.

Across genome backgrounds (H37Rv, L1–4), the simulators successfully produced the target depths and all simulated bases had a quality score above 35 (Figure S1). The simulated AFs show high fidelity to the expected AFs for both simulators and more details are provided in the supplement (Figure S2; Supplementary Results A). The ART-simulated variants demonstrated slightly higher AF variance than the ISS-simulated variants (standard error 0.049% vs 0.031%). All results presented are based on the ISS simulation data. The ART data resulted in similar conclusions and is provided in Supplementary Results E.

When comparing tools, we assessed accuracy as the weighted F1 score pooled over simulated variant AF, sequencing depth and type of region (DR, HT or LM, see Methods) for the H37Rv simulations. We simulated only DR variants in non-H37Rv backgrounds. Accuracy by genomic region was pooled over variant AF and sequencing depth, and accuracy as a function of minimum variant AF was pooled over sequencing depth. BinoSNP is not included in the final tool performance analysis due to high time complexity (see Supplementary Results B).

### FreeBayes achieves the highest overall accuracy

**Fig. 1:**
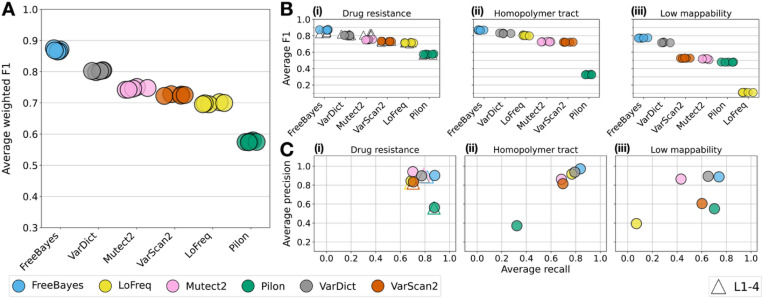
Overall variant caller accuracy. **a** Weighted F1 score achieved by each variant caller in the H37Rv strains, averaged over simulated variant AFs, depths and mutation regions. Each point represents the average weighted F1 score for one of the replicate simulations. **b** Average F1 score achieved by each variant caller in (i) drug resistance, (ii) homopolymer tract, and (iii) low mappability regions. Subplot (i) includes both the H37Rv simulations (colorful circles) and L1–4 simulations (white triangles). **c** Average precision and recall achieved by each variant caller in (i) drug resistance, (ii) homopolymer tract, and (iii) low mappability regions. Subplot (i) includes both the H37Rv simulations (colorful circles) and L1–4 simulations (white triangles with edge colors corresponding to each tool). Note: Pilon does not report INDELs at an AF < 25% by default.

FreeBayes demonstrates the highest average weighted H37Rv F1 score over all mutation types (mean = 0.87, Mann-Whitney P-value = 5.12E-06 for comparison to VarDict; Fig. 1a). VarDict has the next highest weighted F1 score of 0.80, and Pilon achieves the lowest weighted F1 score of 0.58. Across the five genomic backgrounds, all tools except Pilon achieve an average DR-F1 ≥ 0.71 (Pilon average DR-F1 = 0.57; Table S1).

### Low-frequency variant calling accuracy varies by genomic region and is highly uncertain in low mappability regions

Across the three genomic regions, accuracy is lowest and most variable for LM compared with DR or HT regions for all tools except Pilon (LM-F1 is on average 0.22 lower than DR-F1 in the H37Rv samples) (Fig. 1b). FreeBayes achieves either the highest or second-highest precision and recall in each region (Fig. 1c, Tables 1a-c). Tools with the highest overall accuracy demonstrate the lowest variance in performance across the mutation types (DR, HT, LM).

In DR regions, all variant callers achieve comparable H37Rv and L1–4 F1 scores (Fig. 1b(i), Table S1), and the DR-F1 score tool ranking is consistent with the weighted H37Rv F1 score pooled across regions. For each of LoFreq, Mutect2, Pilon and VarScan2, the average DR F1 scores do not differ significantly across the four L1–4 backgrounds (all P-values > 0.05; Figure S3). VarDict and FreeBayes exhibit statistically significant differences in the average F1 score for L3 and every other lineage (P-values ≤ 0.05). The F1 in L3 samples is 0.018 and 0.043 lower than the average F1 across lineages 1, 2 and 4 for FreeBayes and VarDict respectively (see Table S2 and Supplementary Results C).

### Tools demonstrate more than 95% accuracy at AF ≥ 10% outside of low mappability regions

We assessed how the six tools vary in their accuracy as a function of variant AF and sequencing depth. We focused on depths 50–200x as these depths are common in clinical *Mtb* sequencing, and because detection power of low AFs expectedly increases at depths > 200x where tool accuracy also converges (Figure S4). Tool rankings are consistent based on F1 score pooled across all depths and genomic backgrounds, where the average DR F1 > 0.92 for all tools at a variant AF ≥ 5% (Figure S5).

**Fig. 2:**
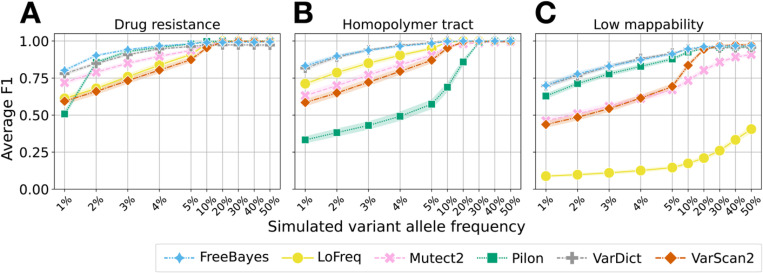
Average cumulative F1 across variant AF pooled over depths of 50x, 100x and 200x. **a** Average cumulative F1 in drug resistance regions (H37Rv and L1–4 strains). **b** Average cumulative F1 in homopolymer tract regions (H37Rv strains only). **c** Average cumulative F1 in low mappability regions (H37Rv strains only). To compute the average cumulative F1, we computed cumulative precision and recall as a function of increasing minimum variant AF for each of the six tools, averaged over haplotype, depths 50–200x and replicate. The band around each line represents the 95% confidence interval. Note that the x-axis tick gaps are not proportional to the actual simulated variant AF, and are larger for AF < 10% as this is where the greatest tool-wise differences occur.

At depths 50–200x and variant AF ≥ 10%, all tools have equivalent cumulative accuracy across DR and HT regions (F1 ≥ 0.95, Fig. 2, Table S3), with the exception of Pilon in HT regions (F1 = 0.69; Pilon does not report INDELs at AF < 25% by default). In LM regions, while FreeBayes, Pilon and VarDict achieve an F1 ≥ 0.93 at variant AF ≥ 10%, VarScan2 and Mutect2 only achieve comparable F1 at variant AFs of 20% and 50% respectively. LoFreq performs poorly in LM regions, achieving an F1 < 0.50 even for variants AF ≥ 50%. Tool rankings are similar for variant calling at AF ≥ 10% and AF < 10%, but across the AF range examined tool accuracy differed the most at lowest AFs (AF 1–5%, Fig. 1, Figure S6).

### Low mappability regions are prone to false positives with high allele frequency

We next determined the number of false positives (FP) detected by each tool by region type and sequencing depth. We expanded the FP assessment to consider LM regions more comprehensively than regions in which we simulated LM low-frequency variants (Supplementary Methods C; “LM” from this point onwards refers to these more comprehensive LM regions). The benchmarking showed strong reference bias, with a false positive rate (FPR) at least fivefold higher in L1–4 than in H37Rv strains for all tools except Pilon (P-values ≤ 0.05; median genome-wide FPR for all tools >7E-05 in L1–4 strains and <1E-04 in H37Rv strains; Figure S7). The two variant callers developed specifically for low-frequency variants, LoFreq and Mutect2, detect the fewest FP per strain (the lowest FPR of 2.90E-05 in an L1–4 strain is achieved by Mutect2). A median of 96% of FPs are SNPs across all tools and genome backgrounds (Figure S8).

**Fig. 3:**
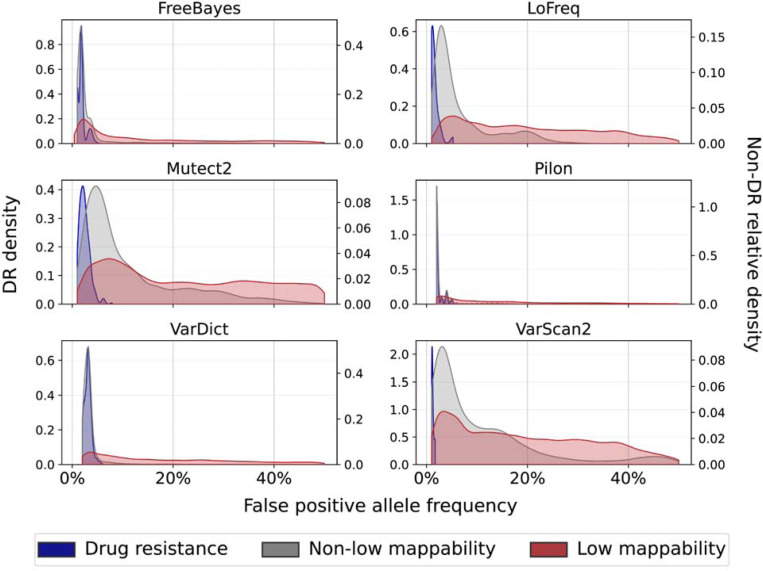
False positive allele frequency distribution in the L1–4 strains by region. Each region is defined to be mutually exclusive for this comparison i.e. the non-low mappability regions do not include the drug resistance regions. The left y-axis displays the densities of the DR AF distributions, and the right y-axis displays the relative densities of the low mappability (non-DR) and non-low mappability (non-DR) AF distributions (normalized independently). For Pilon we include only the FP with AF > 1% (a median of 42% of Pilon FPs across all strains have AF = 1%). All DR FP occur at AF < 8% and FP in the other two regions occur at AFs 1–50%. Between 2–38% of the FP in non-low mappability regions occur at AF > 10%, while more than 60% of the LM FPs occur at AF > 10% for all tools except FreeBayes and Pilon (47% and 50% of the LM FPs have AF > 10% for these tools respectively).

In the L1–4 and H37Rv strains, the FPR is highest in LM regions (median FPR in LM regions is 4.10E-06, versus 0 in DR regions and 1.54E-06 elsewhere in the genome (non-DR and non-LM; Figure S9, Table S4). The average AF for FP LM variants is also significantly higher than for DR or other regions (average AF for LM FPs is 7%, versus 1% for DR and other regions respectively; Fig. 3, Figure S10). Consequently, most of the FP detected in these strains are in LM regions, with the exception of Pilon which detects an excess of FPs at AF = 1% (Table S5). We note that the relationship between FPR and sequencing depth is tool-specific (Figure S11), and the differences in FPR by lineage background (L1–4) are small and detailed in Figure S12.

To better understand sources of FP variant calls we studied read mapping and quality characteristics at variant site positions in the L1–4 strains: including base and mapping quality, coverage relative to the average regional coverage (coverage ratio), the number of discordantly-aligned reads normalized to site coverage and the number of soft-clipped bases normalized to site coverage (Figures S13-17). Mapping quality, coverage ratio, discordantly-aligned reads ratio and soft-clipped bases ratios at a variant site differed significantly between FP and TP variants. There are smaller differences in base quality between FPs and TPs in the simulated data (Figure S1b).

### An error model to improve the specificity of low-frequency variant detection using FreeBayes

FreeBayes had the highest overall performance as measured by average weighted F1, and high performance at AF < 10%. However FreeBayes performance favors higher recall over slightly lower precision for some regions compared with other highly performing tools such as VarDict and Mutect2 (Tables 1a-c). We hypothesized that pairing a high-recall tool like FreeBayes with post-filtering can further improve the balance of recall and precision for low-frequency variant calling.

**Fig. 4:**
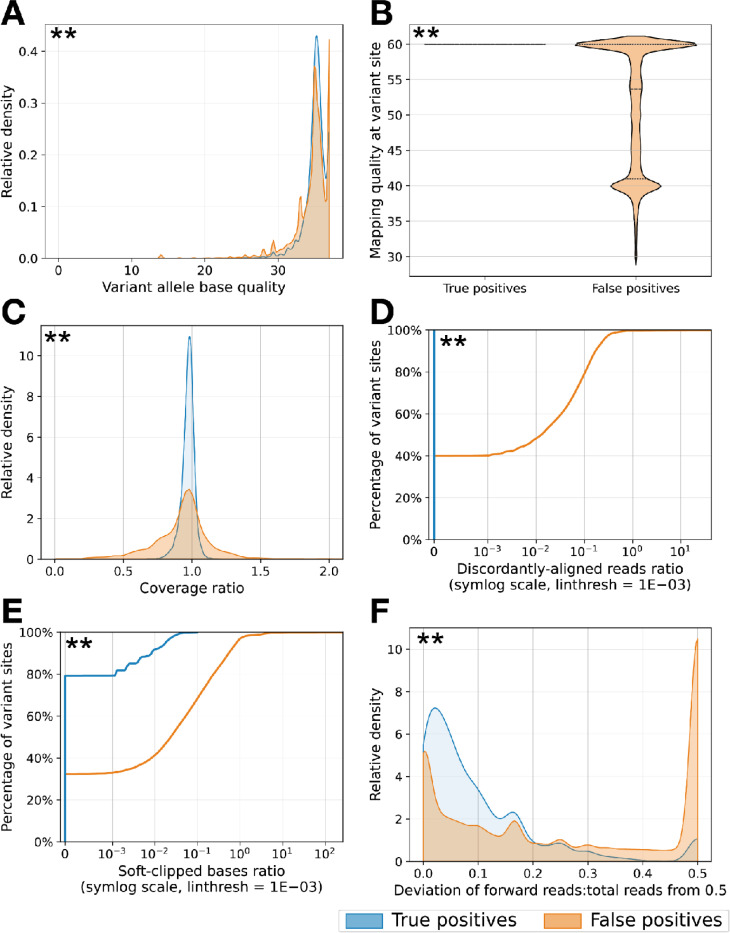
Read mapping and quality characteristics at FreeBayes SNV sites (AF ≤ 50%) split by true and false positives. **a** Base quality. **b** Mapping quality. **c** Coverage ratio: coverage at a variant site relative to the average regional coverage; x-axis is cut-off at 2; 0% of TP sites and 0.09% of FP sites have a coverage ratio > 2. **d** Discordantly-aligned reads ratio: number of discordantly-aligned reads at a variant site relative to site coverage; the empirical cumulative distribution function is shown on a symmetric logarithmic x-axis with a linear threshold of 1E-03; 100% of TP sites and 40% of FP sites have a discordantly-aligned reads ratio of 0. **e** Soft-clipped bases ratio: number of soft-clipped bases at a variant site relative to site coverage; the empirical cumulative distribution function is shown on a symmetric logarithmic x-axis with a linear threshold of 1E-03; 79% of TP sites and 32% of FP sites have a soft-clipped bases ratio of 0. **f** Strand bias: the magnitude of the deviation from 0.5 of the number of forward reads supporting a variant as a fraction of the total reads at the variant site. Mapping quality, coverage ratio, discordantly-aligned reads ratio and soft-clipped bases ratio at a variant site have differing characteristics for FP variant calls compared to TP variant calls. Strand bias and base quality have smaller differences between FPs and TPs. The differences between the TP and FP distributions are statistically significant for each metric after Benjamini-Hochberg correction (indicated by asterisks; Mann-Whitney U test with FDR = 0.05).

We studied the aforementioned five read mapping and quality characteristics (Figures S13-17) in addition to strand bias at FreeBayes SNV call positions (Fig. 4). Given the varied reasons that drive false positive low-frequency variant calls, we used five of the read mapping and quality metrics we studied to build a logistic error model that estimates the probability of a false call for any unfixed SNV with 5% ≤ AF ≤ 95%, at least 2 forward and reverse strand mapped reads, total depth ≥ 5, and MQ ≥ 40. We trained the model on a ground truth set of unfixed SNVs (n=946, 394 true, 552 false) identified by FreeBayes in short-read mapping of 172 *Mtb* isolates to a personal genome built and polished using hybrid long-reads and short-read sequencing (Methods). The model achieves an AUC of 0.929, precision of 0.812 and recall of 0.967 for SNVs on the training set.

We tested this error model on the simulated L1–4 strains to filter FreeBayes SNV calls, focusing on the FP detected with comparable AF to the introduced variants (AF ≤ 50%). The error model excludes, on average, 49% of FreeBayes SNV FPs (AF 5–50%) detected in an L1–4 strain, and <1% of TPs on average per strain (Table 2). Error model filtering combined with hard filtering excludes 65% of FPs, but results in a proportionally higher loss of TPs especially at lower sequencing coverages. With the addition of masking low mappability regions and rRNA genes which naturally excludes all TP calls in these regions, 98% of FPs are filtered per strain on average. An average SNV FPR < 2e-6 is achieved per strain after error model filtering, hard filtering and region masking, more than 100 times lower than the initial FPR. For FreeBayes SNV calls with AF < 5% in the L1–4 strains, model exclusion filters out on average 46% of FPs and <1% of TPs per strain (Table S6).

To filter FP INDEL calls made by FreeBayes we built a pipeline to adjust the allele fraction for unfixed FreeBayes INDEL calls (AF 5–95%) and retain only INDEL calls with AF ≥ 5% post-adjustment (Methods). After allele fraction adjustments for the FreeBayes INDEL calls (AF 5–50%), 43% of the INDEL FPs are filtered out on average per strain for the L1–4 strains (Table S7). Hard filtering on read count thresholds and mapping quality and region masking (see Methods), filters out an average of 99% of the INDEL FPs. Only 6.5% of the total INDEL TPs detected at AF ≥ 5% in the strains simulated from an H37Rv background are lost on average with this filtering scheme, and this loss in sensitivity is more pronounced at lower sequencing coverages (Table S7).

### Tools overestimate allele frequency on average

**Fig. 5:**
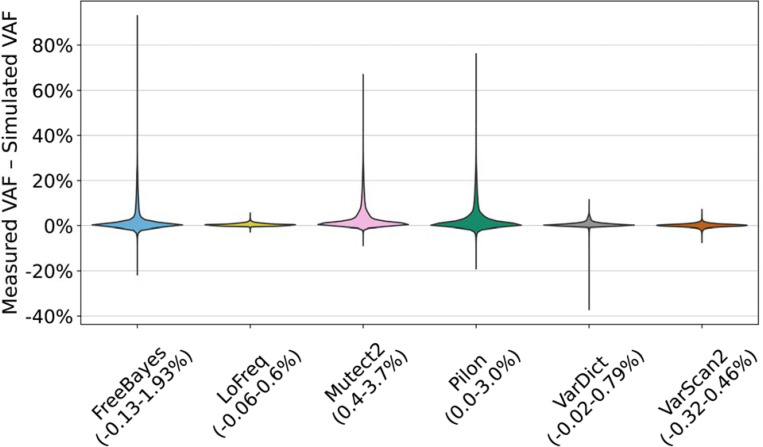
Distribution of the difference between the measured allele frequency and the simulated allele frequency. Each distribution includes these allele frequency differences for variants across all simulated variant allele frequencies, depths, genomic regions, genomic background and replicates. The IQR for each distribution is displayed next to the tool name. VarDict reported 18 variants with an AF at least 35% lower than the simulated AF, six of which were all within ±9 base pairs of each other in the same strain (H37Rv background, sequencing coverage 100x, simulated AF = 40%). All of these variants in the H37Rv strains are in rpoB, which overlaps with our definition of comprehensive LM regions, and 10 of these variants occurred at the same position in gyrA which was found to be problematic for FreeBayes due to its proximity to a baseline (fixed) lineage variant (see Supplementary Results C).

The variant AFs quantified by each tool differ only slightly from the simulated AF as a function of sequencing depth (AF median difference = 0.42% IQR 0–1.3% pooled across tools and depths, Figure S18). Variant callers are more likely to overestimate than to underestimate AF. The AF differences are smallest for LoFreq, VarDict and VarScan2 (median difference for all three tools is between 0–0.34%, Fig. 5, Figure S19). For all three tools, the AF differences of at least 92% of reported variants are within ±2%. FreeBayes, Mutect2 and Pilon have slightly larger differences (median differences are 0.52%, 1.2% and 1.0% respectively), still, 73%, 63% and 60% of the all variant AFs reported by each of these three tools are within ±2% of the simulated value. The larger AF differences for these three tools can be attributed to low-frequency variants in LM regions (Figure S20). The bias in AF measurement does not differ substantially by genomic background in DR regions (median AF difference is 0.24% and 0.26% respectively for H37Rv and L1–4).

### Detection of low-frequency variants in experimental strain mixtures

We compared tool accuracy using sequencing data from six *in-vitro* strain mixtures. The wildtype H37Rv strain that did not harbor a mutation in *rpoB* (SR1a or SR4k) was mixed in proportion with its daughter colony harboring a single *rpoB* mutation (Ser531Leu and His526Pro respectively) generating a final *rpoB* mutation frequency of 1%, 5% or 10%.^40^

**Fig. 6:**
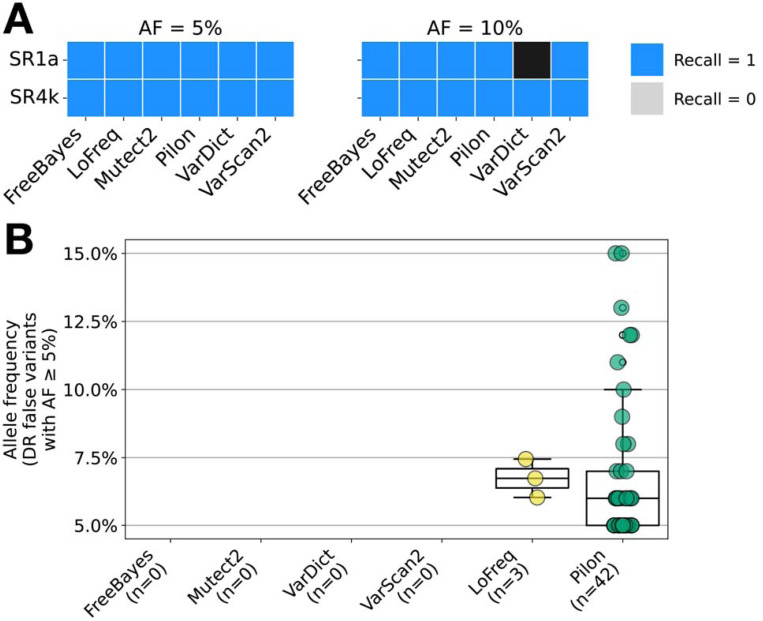
Variant caller performance in in-vitro samples with introduced *rpoB* mutations for AF ≥ 5%. **a** Heatmap of recall for each tool on each in-vitro isolate with rpoB mutations introduced at AF ≥ 5%. VarDict timed out after 3 days for the 10% SR1a isolate. **b** Distribution of the false variant AFs detected by each tool across all DR regions at AF ≥ 5%. The number of total false variants detected at AF ≥ 5% by a tool is shown in parentheses below each tool name.

Five of the six tools successfully detected variants present at AFs of 5% and 10% (Fig. 6a). The exception was VarDict which failed to produce results for one isolate after running for 3 days. Focusing on FPs (variants that were not known to occur in the daughter or wild type strains) at AF ≥ 5%, no FP are detected in *rpoB* by any tool. Outside of *rpoB* in the remaining DR regions, no FPs at AF ≥ 5% were detected except by LoFreq (3 FP across all isolates) and Pilon (42 FP across all isolates) (Fig. 6b). All of the FP detected by LoFreq had AF ≤ 7.5%, while Pilon’s detected FP ranged from AF 5–15%.

Of the 6 tools, only Pilon detected both *rpoB* variants at an AF of 1%, but its precision was low and it falsely identified an average of 87 variants per isolate (AFs 1–4%), 95% of its variants are at AF = 1% (Figure S21b). FreeBayes and VarScan2 were able to identify the AF = 1% *rpoB* variant in the SR1a sample, and both tools also falsely identified an average of 1 variant per isolate.

## Discussion

In this *Mtb*-focused benchmarking analysis, we evaluate a range of factors that influence low-frequency variant calling. Firstly, we consider the genomic context of a variant by comparing the detection of variants in and out of repetitive regions and homopolymeric tracts. Secondly, we study the signal-to-noise ratio by examining the accuracy of variant detection as a function of sequencing depth and within-sample allele frequency. Lastly, we explore reference bias by comparing variant calling in samples simulated from differing background genomes across different *Mtb* lineages and thus, with different levels of baseline genetic divergence from the alignment reference. Across six variant callers, we find the average precision and recall in DR regions to be consistently over 0.92 and 0.82 respectively at a minimum AF of 3% across sequencing depths 50–700x, regardless of background genome. Yet, genome-wide in non-H37Rv strains, a median of more than 300 FP are detected at AF ≥ 1%, with more than a half of these occurring in low mappability regions. This poor precision at low variant AFs, which is reflected in our *in-vitro* analysis, necessitates both a higher minimum variant AF cutoff for genome-wide studies of low-frequency variants, and masking regions of the genome in which variants are inherently harder to detect accurately with short-read sequencing data. Overall, FreeBayes performs consistently well across genomic regions and background genomes, making it a good choice as a broadly applicable tool for both studies of drug resistance and genome-wide within-host diversity.

The high performance of FreeBayes compared to the other tools is possibly due to its haplotype-based variant detection method, as opposed to an alignment-based method. FreeBayes’s local phasing of genotypes into haplotype clusters can prevent variant calls based on poor alignments in low complexity regions, or regions enriched for structural variation, as has been noted in other studies and seen for other haplotype-based tools.^48,51,52^ FreeBayes had not previously been benchmarked on low-frequency variant detection in *Mtb* WGS data, though other studies reported that FreeBayes achieved the highest precision for fixed SNVs and a high recall comparable to 6 out of the 7 other benchmarked tools for fixed SNVs and INDELs in *Mtb* WGS data, and the highest sensitivity at the cost of the lowest precision for minor variants in simulated viral data.^33,48^ Mutect2 is also haplotype-aware, though its precision and recall do not match those achieved by FreeBayes.

VarDict, like Mutect2 and VarScan2, were initially developed for somatic mutation detection. And despite VarDict’s shortcomings with close-proximity variants, all three of these somatic mutation detection tools perform comparably to, if not better than, the other more general-purpose tools we benchmarked. Our study therefore substantiates the use of somatic mutation detection tools for low-frequency variant detection in bacterial data. GATK already encourages this use case with its recent release of a call filter function mode for Mutect2 which fine-tunes it for microbial data. Though LoFreq was developed specifically for low-frequency variant detection in a range of WGS data types, it ranks only above Pilon based on average weighted F1 score. Comparatively, LoFreq is a more conservative variant caller, as observed by others, and exhibits poor performance in repetitive regions of the genome.^30,33^ LoFreq was run with its default mapping quality settings min=0 and max=255, consistent with other studies, but it still achieves poor recall in low mappability regions.^30,33,53^ Pilon is a widely used tool for calling variants in microbial data but we note it was not designed for low-frequency variant reporting.^41^ For example, (1) INDELs occurring at AF < 25% are not reported, and (2) while low-frequency SNVs can be determined from Pilon’s output using the quality weighted percentage (QP) values, we did observe an abundance of false positive variants with AF = 1%, causing uncertainty around the validity of these low (<10) QP values produced by Pilon.

Overall, our analysis necessitates an approach to low-frequency variant detection that (1) imposes sequencing depth-dependent minimum AF thresholds and (2) mitigates false variants in regions of the genome with poor mappability. For all tools, false variants are prevalent at low AFs in DR and other generally easy-to-call regions. Additionally, our analysis of *in-vitro* data showed that all tested variant callers struggled to distinguish between introduced mutations at AF = 1% and false variants, likely caused by factors such as sequencing error and poor mapping. Consequently, we do not recommend calling low-frequency variants at an AF of 1% with short-read sequencing data at standard depths. Further, the effect of reference bias on precision outside of DR regions is substantial and most pronounced in known low mappability regions, where tool performance is also more variable. False variants in these regions are likely due to non-unique mapping between highly similar, repetitive segments of the genome and are observed at AFs 1–50%. Masking these regions in order to mitigate this issue is standard practice in the field, and our analysis shows it to have noticeable benefits on the precision of FreeBayes. Still, the low mappability regions defined in this analysis are not exhaustive, are not all automatically generalizable across organisms, and mask all positions in three *Mtb* DR-associated genes (*rpoB*, *rpoC* and *rrs*) which will impact the clinical relevance of variant discovery.

Our analysis also shows generalizable characteristics of false variants that allowed us to develop an error model for variant filtering that can be used with or without region masking. The model substantially improves the precision of FreeBayes at a low cost to recall. Alternative approaches for accurately detecting variants in these regions include variant calling against a personal or closely related reference genome and/or ultra deep short-read sequencing (500–1000x) or combined short and long-read sequencing.^54–58^ Looking forward, graph-based alignment methods for short-reads can offer further improvements for minority variant calling, especially in structurally divergent regions of the genome.^54,59–61^

Our analysis is not without limitations. We focus strictly on five *M. tuberculosis* genomic backgrounds and examine a subset of variants observed in the *Mtb* population, specifically SNVs and homopolymeric indels. Simulations are naturally reductionist, and do not comprehensively model types of error expected in real-world sequencing data. For example, contamination is an important source of low-frequency variant calls especially in conserved regions of the genome, and we do not examine the effect of contamination on error rates of low-frequency variant detection in this study. Further, we do not investigate recall outside of DR regions in strains simulated from a non-H37Rv background genome.

With these caveats our explicit assessment of mappability and different sources of false positives supports generalizability of our benchmarking exercise to other pathogens and organisms. None of the final six tools discussed in this analysis were developed for *Mtb* specifically, but all achieve practically high and clinically relevant accuracy across high complexity areas of the genome.

## Conclusions

In conclusion, FreeBayes is the top-performing tool for all regions, but low-frequency variant calling is significantly less accurate in low mappability regions. Low-frequency variant detection in DR regions is not affected by reference bias where all tools achieve an average F1 ≥ 0.92 for variant AF ≥ 5% across all background genomes and simulated depths. Our study finds that low mappability region exclusion and minimum variant allele frequencies significantly mitigate high false variant calls genome-wide. Lastly, we provide a new error model that can be used to filter FreeBayes variant calling output reducing low-frequency false positives while maintaining high recall.

## Methods

### Definition

Throughout this study, we use the term low-frequency (minority or unfixed) variants to refer to alleles present within a single *M. tuberculosis* sample at allele frequencies below the consensus threshold of 95%, unless the allele frequency is otherwise specified.

### WGS data simulation

We used InSilicoSeq and ART to simulate paired-end Illumina reads spanning the whole *Mtb* genome. In the first set of simulations, sequencing reads for 500 mutant *Mtb* strains were simulated from the H37Rv reference genome (chromosome NC_000962.3). We considered (1) all 409 SNV DR mutations with known or likely resistance associations reported in the 2021 WHO *Catalogue of mutations in Mycobacterium tuberculosis complex and their association with drug resistance*,^62^ (2) SNV mutations at 46,800 low mappability (LM) positions, and (3) 1-base insertion mutations at 18 homopolymer tracts (HTs) associated with antibiotic resistance or tolerance, for a total of 47,227 potential variant positions (Supplementary Methods A). In each strain we simulated 50 different variants: 20 SNVs at the DR-associated positions, 20 SNVs at the LM positions, and 10 1-base insertion mutations in the HTs with previously known antibiotic resistance or tolerance associations. We generated 10 different haplotypes of these 50 mutations. In the second set of simulations, sequencing reads were simulated from a reference genome belonging to lineage 1 (L1), 2 (L2), 3 (L3), or a non-H37Rv lineage 4 (L4) genome. We simulated 100 strains from each background, for a total of 400 *Mtb* strains, each with 20 simulated DR SNVs. Across both sets of simulations, we varied sequencing depth (50x, 100x, 200x, 400, 700x), and variant allele frequency (1%, 2%, 3%, 4%, 5%, 10%, 20%, 30%, 40%, 50%). Each combination of parameters was simulated in five replicates.

### Non-H37Rv (L1–4) genomes and establishing baseline lineage variants

Reference genomes used for the non-H37Rv simulations were generated by *de novo* PacBio HiFi read assembly and iteratively polished with PacBio and Illumina reads to generate a complete circular genome as described previously.^54^ One sample from each of the lineages 1–4 was selected as the reference genome for the L1, L2, L3 and L4 simulated strains (Additional File 2). We characterized fixed/consensus mutational differences between H37Rv and these reference genomes by aligning each reference to the H37Rv genome with minimap2, and using paftools.js as described by *Marin et al. 2022*.^54,63^ These consensus differences were excluded from analyses assessing false variant detection.

### WGS processing

Each pair of *Mtb* sequencing reads was processed as follows: (1) read trimming from the 3’ end to a quality of 25 with PRINSEQ-lite (v0.20.4),^64^ (2) BWA-MEM (v0.7.17) alignment to the *Mtb* reference genome H37Rv,^65^ (3) duplicate read removal with Picard (v2.8.0),^66^ and (4) mpileup file creation with SAMtools (v1.15.1).^67^

### Variant calling

Seven variant callers were run on the simulated sequence data alignments: BinoSNP, FreeBayes, LoFreq, Mutect2, Pilon, VarDict and VarScan2. For overall analysis we attempted to run each tool with similar or equivalent parameters. For example, for all tools for which we were able, we set the minimum number of alleles required for a variant to be called to 2, as is the default for FreeBayes, VarDict and VarScan2. We also set the minimum variant allele frequency to 0.01 (1%), our minimum simulated variant frequency. When we tested setting the minimum variant allele frequency to 0, some tools picked up significantly more false positives, specifically FreeBayes, which called on average 7,000 more variants in a subset of strains (n=25; Figure S22). For tools without these user-defined parameters, we wrote a script to filter the output of these tools to include only calls with at least 2 variant-supporting reads at a fraction of 0.01 of the coverage at that position. We note that recovery of variants at a frequency of 1% (allele fraction of 0.01) will be limited by the ability of the simulation to accurately simulate enough reads, especially for lower coverages. Ploidy was also set to 1 where possible. Additional variant caller details, parameter tuning and normalization for haplotype-aware tools is described in Supplementary Methods B.

### Real variant analysis

To obtain a baseline expectation for the number of variants in each of the studied regions (DR, HT, LM), we called variants in 38 clinical isolates from the MIC ML Consortium. To estimate the relative number of low-frequency variants in each of the three regions of interest (DR, HT, LM), for each isolate we considered only 1-base insertions in HT regions, and SNVs in DR, LM and all other regions, that were called by *all* six tools (excluding BinoSNP, Table S8). Here, low-frequency variants were defined as variants with average AF (across all tools) between 5% and 95%.

We created two groups from these 38 isolates to investigate differences in the number of variants detected for (1) isolates with difference average sequencing coverages and (2) isolates from different lineages (L1–4). In the first analysis, 20 lineage 4 isolates were studied. Isolates were picked so that there were four isolates with a mean coverage approximately equal to one of the five studied depths (50x, 100x, 200x, 400x, 700x). For the second analysis, six isolates from each of lineages 1–4 were studied. Mean coverage was balanced across all lineage groups and restricted to 50–200x. We also had drug resistance data for 17 drugs; on average, 28% of isolates were resistant for a given drug. The average number of low-frequency variants detected per region was similar between all isolates in each sequencing coverage group and lineage (all P-values > 0.05 for the Mann-Whitney U tests to compare the number of variants in each region between each sequencing depth group and each lineage; Benjamini–Hochberg correction for multiple testing with FDR = 0.05).

### In-vitro isolate analysis

We compared the accuracy of each tool for a set of 6 *in-vitro* isolates each with a single frequently occurring *rpoB* mutation at a frequency of 1%, 5% or 10%. These isolates were prepared by the authors of the binoSNP tool from the *Mtb* reference lab strain (*Mtb* H37Rv ATCC 27294).^40^ A Ser531Leu mutation was introduced in the SR1a isolates, while a His526Pro mutation was introduced in the SR4k isolates. Alignments were prepared and variant calling was performed as described in the *WGS processing* and *Variant calling* methods sections.

### Performance analysis

For each simulated strain we extracted all variants reported by each variant caller and an introduced variant was considered to be present if it occurred in the variant caller output file, regardless of the reported AF. The numbers of ground truth/true positive (TP), false negative (FN) and false positive (FP) variants were determined for each strain. False positive variants were considered at AF ≤ 50%. All tools detected additional variants than the determined baseline lineage variants at AF > 50% in the non-H37Rv strains (median number of these per strain is 77–164 across all tools). These variants are not described as many have very high allele frequency (90–95%) and because a proportion of these are expected due to lineage differences. The latter may have been missed in the baseline lineage variant determination or their call AF determination may have been underestimated by neighboring variants especially for haplotype-based variant callers.

Tool performance in the H37Rv samples was assessed using a weighted F1 score metric. This F1 metric was computed as the harmonic mean precision and recall. The overall precision and recall were computed as the weighted averages of the precision and recall in each of the regions of interest (DR, HT, LM). Weights were defined as the median number of low-frequency variants across 38 clinical isolates for each region as described in the *Real variant analysis* section. We assumed that variant calling accuracy outside of defined DR, HT and LM regions was comparable to accuracy in *Mtb* DR regions in this weighting schema. Precision and recall for the H37Rv samples was determined in all regions (DR, HT, LM). Precision and recall in the L1–4 samples was determined in DR regions only (considering the 20 introduced DR SNVs) and any FP detected in DR regions. Known fixed variants in the L1–4 references relative to H37Rv were excluded.

Accuracy as a function of minimum variant AF (AF 1–50%) was assessed considering all TP, FN and FP with an AF equal to or greater than that minimum variant AF. The per base false positive rate (FPR) per strain was calculated for each region (DR, HT, LM, elsewhere) relative to the region size.

Reported P-values for pair-wise comparisons were obtained using the Mann-Whitney U test (two-sided) and adjusted for multiple testing using the Benjamini-Hochberg procedure to control the false discovery rate. A significance level of 0.05 was used for all tests.

### Linear error model for SNV FP filtering and validation on simulated data

To build a ground truth set of unfixed SNVs and indels, we used hybrid assemblies constructed from short- and long-reads generated for 172 *Mtb* samples. PacBio HiFi reads were assembled using 3 iterations of flye (v2.9.2),^68^ circularized with circlator (v1.5.5),^69^ and then polished with Illumina reads using Pilon (v1.23),^41^ generating “personal reference genomes.” The Illumina reads were preprocessed using fastp (v1.0.1) for adapter trimming and removal of reads shorter than 50 base pairs,^70^ and only reads mapped to the MTBC (taxid 77643) and its lineage by kraken2 (v2.1.3) using the standard database (downloaded in June 2020) were retained.^71^

To call low-frequency variants from the personal reference genomes, we aligned Illumina reads from the previous step to the personal genome using BWA MEM (v0.7.19) with a seed length of 80.^65^ We then marked duplicates with Picard (v3.4.0) and performed variant calling with FreeBayes (v1.3.10) using minimum mapping quality = 30, minimum base quality = 30, minimum alternate allele count = 2, and minimum allele fraction = 0.01.^44,66^ Unfixed variants were transferred from the personal genome coordinates to H37Rv coordinates using *paftools liftover*, which is part of the minimap2 suite (v2.30).^63^ The resulting variants made up the set of real unfixed variants.

The same Illumina reads were aligned to the H37Rv reference genome (NCBI RefSeq NC_000962.3), followed by variant calling using the same tools, versions, and parameters as above for the personal reference genomes. SNVs detected from H37Rv with a within-sample allele fraction ≥0.05 and ≤0.95 were passed into a mixed effects logistic model implemented in R (v4.4.2) using the lme4 package. The five fixed effects in the model were the number of discordantly paired reads normalized to coverage, number of clipped bases normalized to coverage, average base quality of bases supporting the variant, ratio of coverage at the site to the rolling average, and the absolute value of the difference between 0.5 and the proportion of reads supporting the variant that are in the forward orientation. The rolling average of coverage was computed with a window size of 100 base pairs, and we took the maximum of the rolling averages computed from the left and right directions.

The error model was trained on 946 low-frequency SNV calls (394 real, 552 not real) from the 172 WGS samples with matched personal reference genomes. The 552 false variants were detected when aligning Illumina reads to H37Rv, but not when the same reads were aligned to the personal reference genomes. The single random effect was the sample of origin because all variants from all samples were pooled into a single model, and therefore, the predictions for the 3,357 candidate variants in samples not in the training set could only be made based on the estimated fixed effects. After fitting the model, the classification threshold for dichotomizing predicted probabilities was selected to be 0.46 to maximize the sum of precision and recall on the training set.

For each simulated strain, we filtered the set of FreeBayes variants to include only SNPs, extracted the metrics used as fixed effects in the model for each variant, fit the model and retained only variants with a predicted probability > 0.46. We explored the reduction of false variants and loss of true variants after (1) error model filtering, (2) error model and hard filtering (forward and reverse strand allele counts ≥ 2, depth ≥ 5, mapping quality ≥ 40), and (3) error model filtering, hard filtering and low mappability region and rRNA gene masking.

### Adjusting allele fractions to correct unfixed indels

Calling low-frequency indels is generally less affected by reference bias because variant callers require greater evidence to call indels than SNVs. The primary issue in accurately calling low-frequency indels is reads not sufficiently covering an indel. These reads artificially bring down the allele fraction of the indel, making it appear as though a consensus indel is low-frequency and inflating the number of low-frequency indels when simply thresholding on allele fraction.

After variant calling using FreeBayes using the same parameters as for SNVs, indels were left-aligned and normalized using bcftools (v1.21).^72^ For each indel with an allele fraction 0.05–0.95 (inclusive), we then extracted all the reads from the pileup at the position where the indel begins. We excluded all reads with soft clipping and all reads that start or end within 10 base pairs of the putative indel, including reads that terminate in the middle of the indel. We then determined if the start and end positions for reads that support the indel are significantly different from those of reads that do not support any indel. We compared the medians of the distributions using two Mann-Whitney U tests, one for the start positions and one for the end positions. If both tests returned P-values < 0.01, then this was considered evidence that the reads that do not support the indel do not sufficiently cover it. These reads would be unable to support an indel, even if it truly exists, and so they too were excluded. We then recomputed the allele fraction using all remaining reads that do and do not support the indel (if both P-values from the Mann-Whitney U tests were < 0.01, then these two values are equivalent, and the new allele fraction is 1).

For each simulated strain, we filtered the set of FreeBayes variants to include only INDELs, adjusted the allele fractions for these INDEL variants as described above, and retained only variants with an adjusted allele fraction above 0.05. We explored the reduction of false variants and loss of true variants after (1) allele fraction adjustment, (2) allele fraction adjustment and hard filtering (forward and reverse strand allele counts ≥ 2 for any INDEL except deletions >10bp, depth ≥ 5, mapping quality ≥ 40, adjusted INDEL-supporting reads ≥ 5, variant site coverage ≥ one-third of the genome-wide median), and (3) allele fraction adjustment, hard filtering and masking of low mappability regions, rRNA genes and regions within 100bp of insertion sequences or phages.

## Supplementary Material

Additional File 1 **(.pdf)** Supplementary text, figures and tables.
In this document we describe all supplementary results referenced in the manuscript: (**A**) Simulated allele frequencies show high fidelity to the expected allele frequencies, (**B**) BinoSNP runs inefficiently and detects many of the same false positives as the other variant callers, (**C**) Variant caller failure to detect simulated variants in close proximity to fixed baseline lineage variants, (**D**) FreeBayes and Pilon detect an excessive number of false positives at specific simulated sequencing depths, (**E**) Alternative WGS data simulator comparison: variant caller accuracy rankings and major FP trends are consistent with the ISS-simulated data. We also elaborate on our methods: (**A**) Variant position choices for simulations, (**B**) Variant caller parameters, normalization and filtering, (**C**) Comprehensive low mappability regions.Finally, we provide all supplementary figures (Figures S1-34) and tables (Tables S1-9).

Additional File 2 **(.xls)** Reference genome information for the L1–4 simulations.
This Excel spreadsheet details the run accession numbers associated with the sequencing reads used to generate the L1–4 reference genomes. The L1–4 reference fasta files are available for download here: https://zenodo.org/records/13761165.

## Figures and Tables

**Table 1a:** Average DR precision and recall in the strains simulated from an H37Rv and L1–4 background.

Rank	Drug resistance precision				Drug resistance recall			
	H37Rv		L1–4		H37Rv		L1–4	
	Variant caller	Average precision	Variant caller	Average precision	Variant caller	Average recall	Variant caller	Average recall
1	Mutect2	0.941*	Mutect2	0.918*	FreeBayes	0.879	Pilon	0.873*
2	FreeBayes	0.902*	VarDict	0.891*	Pilon	0.876*	FreeBayes	0.819*
3	VarDict	0.899*	FreeBayes	0.886*	VarDict	0.772	Mutect2	0.791*
4	LoFreq	0.843	LoFreq	0.824	VarScan2	0.706*	VarDict	0.785
5	VarScan2	0.834*	VarScan2	0.822*	Mutect2	0.701*	VarScan2	0.704
6	Pilon	0.564	Pilon	0.559	LoFreq	0.687	LoFreq	0.683

Tools are ranked according to precision and recall separately for each background genome group. Asterisks indicate the precision and recall rankings that are statistically significant after Benjamini-Hochberg correction (Mann-Whitney U test with FDR = 0.05, e.g. in the first row of the precision section of the table we tested for a significant difference in the distribution of precision values between the first and second ranking ranking tools).

**Table 1b:** Average HT precision and recall in the strains simulated from an H37Rv background.

Rank	Homopolymer tract precision		Homopolymer tract recall	
	Variant caller	Average precision	Variant caller	Average precision
1	FreeBayes	0.972*	FreeBayes	0.837*
2	VarDict	0.937*	VarDict	0.793
3	LoFreq	0.916*	LoFreq	0.769*
4	Mutect2	0.862*	VarScan2	0.696*
5	VarScan2	0.815*	Mutect2	0.684*
6	Pilon	0.371	Pilon	0.322

Tools are ranked according to precision and recall separately. Asterisks indicate the precision and recall rankings that are statistically significant after Benjamini-Hochberg correction (Mann-Whitney U test with FDR = 0.05).

**Table 1c:** Average LM precision and recall in the strains simulated from an H37Rv background.

Rank	Low mappability precision		Low mappability recall	
	Variant caller	Average precision	Variant caller	Average precision
1	VarDict	0.893*	FreeBayes	0.740*
2	FreeBayes	0.887*	Pilon	0.703*
3	Mutect2	0.864*	VarDict	0.652*
4	VarScan2	0.605	VarScan2	0.602*
5	Pilon	0.551*	Mutect2	0.432*
6	LoFreq	0.394	LoFreq	0.070

Tools are ranked according to precision and recall separately. Asterisks indicate the precision and recall rankings that are statistically significant after Benjamini-Hochberg correction (Mann-Whitney U test with FDR = 0.05).

**Table 2:** SNV filtering of false and true variants called by FreeBayes (AF 5–50%) in L1–4 strains.

Sequencing coverage	Total		Percentage filtered					
	Pre-filtering		F1: Error model filtering		F2: Error model + hard filtering		F3: Error model + hard filtering + region masking	
	FP	TP	FP	TP	FP	TP	FP	TP
50x	55492	2012	56.4%	0.2%	75.8%	20.6%	98.9%	24.6%
100x	55366	2017	53.4%	0.0%	70.2%	3.8%	98.6%	9.3%
200x	54980	2063	48.0%	0.0%	63.9%	0.1%	98.3%	5.2%
400x	54813	2045	43.4%	0.0%	59.1%	0.0%	98.2%	5.7%
700x	53920	2029	42.5%	0.0%	58.0%	0.0%	98.2%	5.2%
Overall	274571	10166	48.7%	0.04%	65.4%	4.9%	98.4%	10.0%

The pre-filtering group of columns displays the total number of FP and TP SNVs called by FreeBayes across all strains simulated at a specific sequencing depth. A total of 120 strains are considered in each sequencing depth group (six variant AFs 5–50%, four background genomes, five replicates). The F1-F3 groups of columns correspond to each of the three sequential SNV filtering schemes and display the average percentages of FPs and TPs filtered out per strain. F1 (Filter 1): error model filtering, F2: error model filtering and hard filtering (forward and reverse strand allele counts ≥ 2, depth ≥ 5, mapping quality ≥ 40), F3: error model filtering, hard filtering and region masking (low mappability regions and rRNA genes). FP and TP statistics are broken down by simulated sequencing coverage group, and summarized overall. Note for F3 that we simulated variants in the regions masked in this filtering scheme.

## Data Availability

All code for data processing and analysis in this study is available from the following GitHub repository, https://github.com/shandu-m/benchmark-minority-variants-Mtb.
