# Supplementary material for: Benchmarking within-sample minority variant detection with short-read sequencing in M. tuberculosis
Source: bioRxiv. 2026 Feb 16:2026.02.13.704885. Preprint. [Version 1] doi: 10.64898/2026.02.13.704885 (PMC12934614; doi:10.64898/2026.02.13.704885)
Supplement: Additional File 1 — (.pdf) Supplementary text, figures and tables. In this document we describe all supplementary results referenced in the manuscript: (A) Simulated allele frequencies show high fidelity to the expected allele frequencies, (B) BinoSNP runs inefficiently and detects many of the same false positives as the other variant callers, (C) Variant caller failure to detect simulated variants in close proximity to fixed baseline lineage variants, (D) FreeBayes and Pilon detect an excessive number of false positives at specific simulated sequencing depths, (E) Alternative WGS data simulator comparison: variant caller accuracy rankings and major FP trends are consistent with the ISS-simulated data. We also elaborate on our methods: (A) Variant position choices for simulations, (B) Variant caller parameters, normalization and filtering, (C) Comprehensive low mappability regions.Finally, we provide all supplementary figures (Figures S1-34) and tables (Tables S1-9). [file media-1.pdf]

Supplemental Information

Benchmarking within-sample minority variant detection with short-read sequencing in *M. tuberculosis*

Shandukani Mulaudzi[1,*], Sanjana Kulkarni[1], Maximillian G. Marin[1,2], Maha Farhat[1,3,*]

[1]Department of Biomedical Informatics, Harvard University, Boston, MA 02115, USA

[2]Department of Data Science, Dana-Farber Cancer Institute, Boston, MA 02215, USA

[3]Division of Pulmonary and Critical Care, Department of Medicine, Massachusetts General Hospital, MA 02114, USA

[*]Corresponding authors: smulaudzi@g.harvard.edu, maha_farhat@hms.harvard.edu

Supplemental Information Table of Contents

Supplementary Results 3
A. Simulated allele frequencies show high fidelity to the expected allele frequencies 3
B. BinoSNP runs inefficiently and detects many of the same false positives as the other variant callers 3
C. Variant caller failure to detect simulated variants in close proximity to fixed baseline lineage variants 4
D. FreeBayes and Pilon detect an excessive number of false positives at specific simulated sequencing depths 4
E. Alternative WGS data simulator comparison: variant caller accuracy rankings and major FP trends are consistent with the ISS-simulated data 4

Supplementary Methods 6
A. Variant position choices for simulations 6
B. Variant caller parameters, normalization and filtering 7
C. Comprehensive low mappability regions 10
References 11

Supplementary Figures 13

Supplementary Tables 50

Supplementary Results

A. Simulated allele frequencies show high fidelity to the expected allele frequencies

The standard error of the simulated AFs for ISS and ART was respectively 0.011% and 0.015% for AF < 10%, and respectively 0.051% and 0.084% for AF ≥ 10% (averaged across all depths and background genomes). Most of the variability in the simulated AFs for each simulator tool can be attributed to the higher standard error of the simulated AFs in LM regions, and the simulated LM variant AF is consistently lower than the expected AF across all depths (Figure S23, Table S9). For both ISS and ART, the simulated values are consistent across all replicates (all P-values > 0.001 for replicate comparisons of simulated coverage and AF in each expected sequencing coverage group, and simulated base quality).

B. BinoSNP runs inefficiently and detects many of the same false positives as the other variant callers

BinoSNP requires a user to supply it a BED file with regions in which to look for mutations and a table with an entry for each position in these regions specifying the reference and alternate allele. We tested BinoSNP on a subset of 500 strains (one of the replicates), supplying input regions of length 10^1, 10^2, 10^3, 10^4, 10^5 and 10^6. With a region size of 10^5, the run times were already well above 10 hours per strain, while the strain runs with a region size of 10^6 timed out after 5 days (Figure S24). The BinoSNP tool offers no parallelization, though it would be possible to split the input region files and call variants for a strain in multiple regions concurrently. We do find, however, that BinoSNP is not able to filter out FP variants detected by other tools successfully. For each of the strains simulated by InSilicoSeq (n=2,500), we provided BinoSNP with all positions at which a variant had been detected by at least one of the other variant callers and determined the percentage of FPs detected by at least one of the other variant callers at AF > 1% that was also detected by BinoSNP. Across all strains, BinoSNP detects an average of 85% of the FPs detected by at least one other variant caller (minimum = 33%), and this percentage range is similar across each subset of strains simulated with a different sequencing depth (84-88%). BinoSNP displays no bias towards genomic region, detecting an average of 85-90% of the FPs per strain detected by at least one other variant caller at AF > 1% in all regions of the genome outside of HT regions. In HT regions, BinoSNP detected only 7% of the FPs detected by at least one other variant caller, though in preliminary analysis, we found the INDEL reporting of BinoSNP to be unreliable.

C. Variant caller failure to detect simulated variants in close proximity to fixed baseline lineage variants

FreeBayes consistently missed one mutation in L1, L2 and L4 samples, and two mutations in L3 samples, and VarDict consistently missed two mutations in L3 samples (Figure S25). These shortcomings in the recall of FreeBayes and Vardict were linked to three consistently missed mutations across all simulated L1-4 strains, all three of which were outside of the LM regions. FreeBayes missed the *gyrA* mutation at position 7,582 in at least 94% of strains in each lineage group, as well as the *pncA* mutation at position 2,289,050, which was missed in 98% of L3 strains. Both of these mutations were found to occur within 3bp of a lineage variant (Figure S26). This proximity to a lineage variant was able to differentiate both of these mutations that were consistently missed by FreeBayes from the other mutations that FreeBayes did not consistently miss (Figure S27a). While VarDict also missed the *pncA* mutation at position 2,289,050 in 98% of L3 strains, it additionally missed a *pncA* mutation at position 2,289,072 in 76% of L3 strains. The mutation at position 2,289,072 was not as close to any L3 lineage variants, or other simulated mutations, as the other two consistently missed mutations. This mutation could not be differentiated from other unproblematic mutations for any base pair window size (Figures S27b-d).

D. FreeBayes and Pilon detect an excessive number of false positives at specific simulated sequencing depths

Both FreeBayes and Pilon exhibited an unexpected excess number of FPs at a subset of the tested sequencing depths: most significantly at 100-200x for FreeBayes and 200-400x for Pilon (Figure S11). This pattern is present in the ART data as well (Figure S28). Using a set of 20 clinical isolates, with average sequencing depths distributed across our simulated depths, we investigated the total number of variants found by each tool in each depth group (Figure S29). We discovered a similar pattern as exists in the simulated data: FreeBayes found an excess of variants at depths of 100-200x, while Pilon found an excess of variants at depths of 200-400x.

E. Alternative WGS data simulator comparison: variant caller accuracy rankings and major FP trends are consistent with the ISS-simulated data

FreeBayes still achieves the highest accuracy in the ART H37Rv simulations according to weighted F1 score (Fig. 1a, Figure S30a). While Pilon still achieves the lowest accuracy too, the two pairs of tools achieving intermediate weighted F1 scores are swapped (Mutect2-VarDict and LoFreq-VarScan2). All variant callers perform consistently between the ISS and ART

simulations in DR and HT regions (average F1 score difference ART-ISS < ±0.03 in both regions), performance in LM regions is lower overall (average F1 score difference ART-ISS = -0.34) and variant caller ranking is significantly shuffled (Fig. 1b, Figure S30b). Consistent with the ISS simulations, variant caller F1 scores are most variable for variant AF < 10% where FreeBayes achieves the highest F1, and LoFreq, VarDict and VarScan2 most successfully recapitulate simulated variant AF (Figures S31, S32).

While fewer FPs are detected by all tools in the ART data than in the ISS data (1.45E-05 and 2.21E-05 median FPR respectively across all strains and tools), a higher FPR is still observed in LM regions than in DR regions or elsewhere in the genome (Figures S9, S33). Additionally, FPs in DR regions remain at low AFs <10.2% in the ART data (Fig. 3, Figure S34). Finally, though low mappability regions are still prone to high AF FPs in the ART data, there are more high AF FPs outside of LM regions in the ART data than in the ISS data.

Supplementary Methods

A. Variant position choices for simulations

i. SNVs in drug resistance regions

SNV positions for each *Mtb* mutant are randomly chosen from the list of category 1 or 2 confidence level SNVs reported by the WHO (variants with final confidence gradings for resistance associations of "Assoc w R - Interim" or "Assoc w R").[1]

For our analysis of false positives, we considered a broader definition of drug resistance regions. These were based on gene set categorizations from *Vargas et al. 2021*.[2]

iii. Insertions in homopolymer tracts

Homopolymer tract (HT) regions came from the list of regions with a single nucleotide repeated ≥7 times generated by *Vargas et al. 2023* in Supplementary Data File 3.[3] From these HT regions, we included only those with previously known antibiotic resistance/tolerance associations, or reported to have such an association by *Vargas et al*.[4] We also included the 6-guanine homopolymer in *Rv0678* (at position 193) in which spontaneous G-insertions and G-deletions at position 193 were observed in a study on *Mtb* clinical isolates and associated with low-level bedaquiline resistance.[5] Frameshifts in this HT have been observed in several other studies on *Mtb* clinical isolates.[6–9]

For analysis of false positives, mutations in all HT regions were considered (not only those with antibiotic resistance/tolerance associations).

iii. SNVs in low mappability regions

Low mappability regions were defined based on pileup mappability scores, a metric of how easy it is to align reads uniquely across the genome. To determine these scores, the pupmapper pipeline (https://github.com/maxgmarin/pupmapper/) was run on the H37Rv genome. This pipeline determines a pileup mappability score for each genomic position based on k-mer uniqueness up to a defined edit distance. We used pupmapper with a k-mer size of 50bp and a maximum mismatch threshold (edit distance) of 4bp. In addition, we included genomic positions with a low empirical base-level recall (EBR).[10]

A low mappability region for our purposes is defined as a feature (gene or intergenic region) in which all positions have a pileup mappability score < 0.8 and EBR < 0.8. We focus on PE/PPE genes, which are often excluded in analyses due to their repetitive regions, in addition to intergenic regions. Positions in these regions were then randomly selected for each simulated mutant strain.

B. Variant caller parameters, normalization and filtering

BinoSNP (v1.0.1)

BinoSNP was run with its default parameters with a P-value of 0.05 used to filter variants.

FreeBayes (v1.3.6)

For main performance analysis, FreeBayes was run with its default parameters except for our settings of ploidy (1) and minimum alternate fraction (0.01), as well as the settings of minimum mapping quality (30) and minimum base quality (30) as is used in *Nimmo et al. 2019* in which FreeBayes is used to call low-frequency variants in *Mtb* sputum samples.[11] FreeBayes outputs a quality score for each putative variant and suggests filtering variants with this metric on its GitHub page (https://github.com/freebayes/freebayes). In preliminary analysis on simulated data to determine an appropriate quality score threshold for variant filtering, we found overlapping quality score distributions for true positive and false positive variants. As a result, variants called by FreeBayes were not filtered on quality scores for the simulated data analysis.

LoFreq (v2.1.5)

LoFreq was run in its default form to support indel calling: *lofreq indelqual* was used to insert indel qualities into the input BAM file and the resulting BAM file was run through *lofreq call-parallel*. The default P-value of 0.01 was used after testing LoFreq on a subset of simulated data to determine the optimal value (results were consistent across the data simulated by both InSilicoSeq and ART).

Mutect2 (GATK v4.5.0.0)

Mutect2 was run following the GATK best practices documentation for somatic short variant discovery (https://gatk.broadinstitute.org/hc/en-us/articles/360035894731-Somatic-short-variant-discovery-SNVs-Indels), excluding the contamination determination step as we ran all tools on simulated data: candidate variants were called using Mutect2, orientation bias artifacts were determined using *LearnReadOrientationModel*, and putative variants were labeled with *FilterMutectCalls* in microbial mode. In the *FilterMutectCalls* step, calls are assigned labels such as “PASS”, “orientation”, “weak_evidence” and “strand_bias.” Typically only “PASS” calls are used for downstream analysis. We found, however, that the “orientation” label seemed to be inflated in the simulated data in a way that was not reflective of true error. Further, for the InSilicoSeq simulated data, we found that the average true positive rate increased by 0.447, while the

average false positive rate only increased by less than 1E-09 when “orientation” labeled calls were included. An average true positive rate increase of only 0.026 was observed for the ART simulated data (average false positive rate only increased by less than 1E-10), indicating that Mutect2 was more sensitive to orientation bias in the InSilicoSeq data. The “orientation” labels were not prevalent in our cohort of clinical isolates used in this study (<1% of variants called by all tools were labeled with “orientation” by *FilterMutectCalls*), further substantiating that the “orientation” over-labeling of true positives by *FilterMutectCalls* is a simulation-specific issue.

Pilon (v1.24)

For main performance analysis, Pilon was run with the same parameter settings used by *Marin et al. 2022* in a paper benchmarking the empirical accuracy of short-read sequencing in *Mtb*: minimum mapping quality and minimum depth were set to 40 and 5 respectively.[10] The variant allele read count was calculated with QP and DP Pilon VCF output fields, *(QP/100)*DP*. The Pilon output VCF was filtered to include all positions at which there were at least 2 high quality reads supporting the variant allele and at which the quality-weighted support for that base was at least 1 (corresponding to an allele frequency of 1%).

VarDict (Java port v1.8.3)

VarDict was run in its default form which includes feeding its output through the *testsomatic.R* and *var2vcf_paired.pl* scripts for paired variant calling, as described by VarDict’s GitHub page (https://github.com/AstraZeneca-NGS/VarDict). The VarDict output VCF was filtered to include all positions at which there were at least 2 reads supporting the variant allele and at which the allele frequency for that base was at least 1%.

VarScan2 (v2.3)

VarScan2 tools *mpileup2snp* and *mpileup2indel* were run with minimum variant frequency set to 0.01 (1%) and their outputs were combined into one TSV file. A P-value of 0.01 was also used after testing VarScan2 on a subset of simulated data to determine the optimal value (InSilcoSeq and ART results were consistent) and supported by literature.[12] Otherwise, all VarScan2 parameters were set to their default.

Variant set normalization and filtering

To ensure accurate comparison of variants called between tools and to the introduced variants, we took steps to standardize the variant output across all tools. We used the *norm --multiallelics*

-any function (with a reference genome fasta file) from bcftools (v1.21) to split multi-alleleic sites and left-align INDELs in the FreeBayes, VarDict and Mutect2 output VCF files.[13] The *vcfwave* tool from vcflib (v1.0.14) was additionally used to simplify complex alleles called by FreeBayes and VarDict.[14] MNPs called by FreeBayes, VarDict and Mutect2 were split into SNPs in a custom Python script. Finally, for all tools we used a custom Python script to extract variant calls from the tool output VCF or TSV file and perform the necessary filtering.

C. Comprehensive low mappability regions

We expanded our definition of low mappability regions for analysis beyond the low mappability regions used as candidate variant simulation positions. These regions are based on a range of factors including pileup mappability scores, or determined through empirical studies of the *Mtb* genome in work within our lab and with collaborators.

These comprehensive low mappability regions include:

1. Regions of low pileup mappability determined by pupmapper (https://github.com/maxgmarin/pupmapper/) in H37Rv and 4 other representative genomes from lineages 1-4.
2. minimap2-based H37Rv homologous regions, which allow for indels between homologous sequences.[15]
3. A set of refined low confidence (RLC) regions, describing regions that account for major sources of error in Illumina WGS analysis, determined in *Marin et al. 2022*.[10]
4. Genes with high homology to other bacterial genomes (*aspT*, *clpB*, *hsp*, *rpoB*, *rpoC*, *rpsC*, *rrl*, *rrs*, *tuf*).
5. The first 500bp of the genome which are prone to clusters of erroneous low-frequency variants due to poor alignment and generally lower coverage (in *dnaA*).

Cumulatively, these regions account for 10.6% of the *Mtb* genome.

References

1. *Catalogue of Mutations in Mycobacterium Tuberculosis Complex and Their Association with Drug Resistance*. (World Health Organization, Geneva, 2021).
2. Vargas, R. *et al.* In-host population dynamics of Mycobacterium tuberculosis complex during active disease. *eLife* **10**, e61805 (2021).
3. Vargas, R. *et al.* Phase variation as a major mechanism of adaptation in Mycobacterium tuberculosis complex. *Proc. Natl. Acad. Sci.* **120**, e2301394120 (2023).
4. Safi, H. *et al.* Phase variation in Mycobacterium tuberculosis glpK produces transiently heritable drug tolerance. *Proc. Natl. Acad. Sci.* **116**, 19665–19674 (2019).
5. Xu, J. *et al.* Bedquiline Resistance Mutations: Correlations with Drug Exposures and Impact on the Proteome in M. tuberculosis. *Antimicrob. Agents Chemother.* **67**, e01532-22 (2023).
6. Peretokina, I. V. *et al.* Reduced susceptibility and resistance to bedaquiline in clinical M. tuberculosis isolates. *J. Infect.* **80**, 527–535 (2020).
7. Villellas, C. *et al.* Unexpected high prevalence of resistance-associated Rv0678 variants in MDR-TB patients without documented prior use of clofazimine or bedaquiline. *J. Antimicrob. Chemother.* **72**, 684–690 (2017).
8. Ismail, N., Omar, S. V., Ismail, N. A. & Peters, R. P. H. Collated data of mutation frequencies and associated genetic variants of bedaquiline, clofazimine and linezolid resistance in Mycobacterium tuberculosis. *Data Brief* **20**, 1975–1983 (2018).
9. Kadura, S. *et al.* Systematic review of mutations associated with resistance to the new and repurposed Mycobacterium tuberculosis drugs bedaquiline, clofazimine, linezolid, delamanid and pretomanid. *J. Antimicrob. Chemother.* **75**, 2031–2043 (2020).
10. Marin, M. *et al.* Benchmarking the empirical accuracy of short-read sequencing across the *M. tuberculosis* genome. *Bioinformatics* btac023 (2022) doi:10.1093/bioinformatics/btac023.

11. Nimmo, C. *et al.* Whole genome sequencing Mycobacterium tuberculosis directly from sputum identifies more genetic diversity than sequencing from culture. *BMC Genomics* **20**, 389 (2019).

12. Mariner-Llicer, C. *et al.* Genetic diversity within diagnostic sputum samples is mirrored in the culture of Mycobacterium tuberculosis across different settings. *Nat. Commun.* **15**, 7114 (2024).

13. Danecek, P. *et al.* Twelve years of SAMtools and BCFtools. *GigaScience* **10**, giab008 (2021).

14. Garrison, E., Kronenberg, Z. N., Dawson, E. T., Pedersen, B. S. & Prins, P. A spectrum of free software tools for processing the VCF variant call format: vcflib, bio-vcf, cyvcf2, hts-nim and slivar. *PLOS Comput. Biol.* **18**, e1009123 (2022).

15. Li, H. Minimap2: pairwise alignment for nucleotide sequences. *Bioinformatics* **34**, 3094–3100 (2018).

Supplementary Figures

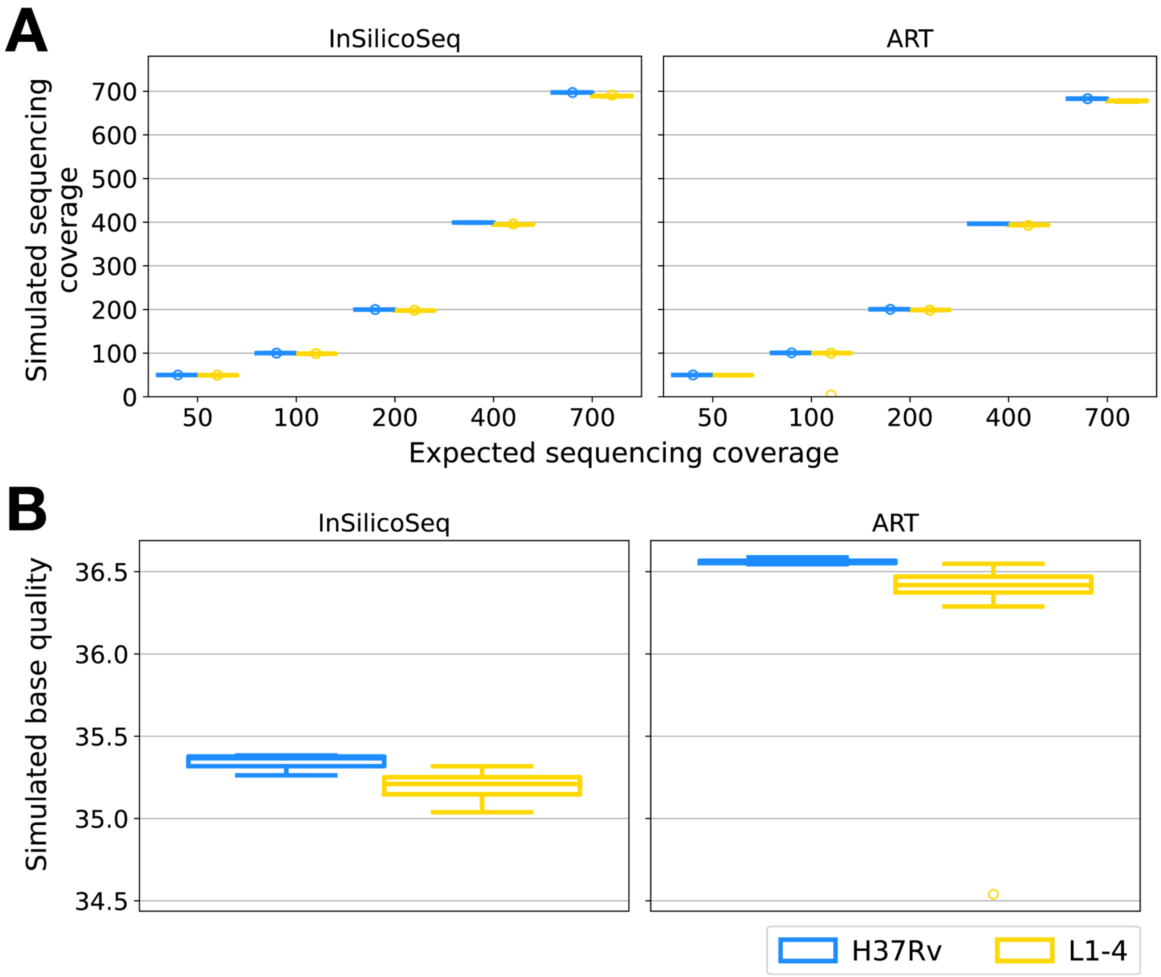

Figure S1 Simulated sequencing coverage and base quality achieved by InSilicoSeq (ISS) and ART. The coverage and base quality are displayed for the strains simulated from H37Rv and those simulated from an L1-4 background genome separately. **a** Simulated sequencing coverage. The differences in the average sequencing coverage between the H37Rv and L1-4 strains are similar for the ISS and ART simulations and minimal for both (average H37Rv – L1-4 coverage for ISS and ART respectively is 0.56, 0.37 at 50x, and 7.83, 5.18 at 700x where this difference is highest across all expected sequencing depths. **b** Simulated base quality. All average strain base qualities were within 35.03-35.39 for the ISS simulation, and within 34.53-36.59 for the ART simulation. The outlier with an average depth ~ 0 in the ART L1-4 100x strain group (average simulated depth = 4.2x) and outlier with an average base quality under 35.0 in the ART L1-4 simulation group (average base quality = 34.5) are the same strain. Both the simulated depth and simulated base quality were determined by running Pilon (as described in Appendix B) on each simulated strain to produce a VCF entry for each genomic position. The DP (depth) and BQ (base quality fields) were then extracted for each position and averaged for each strain. The distribution of these averages is displayed in the plots above.

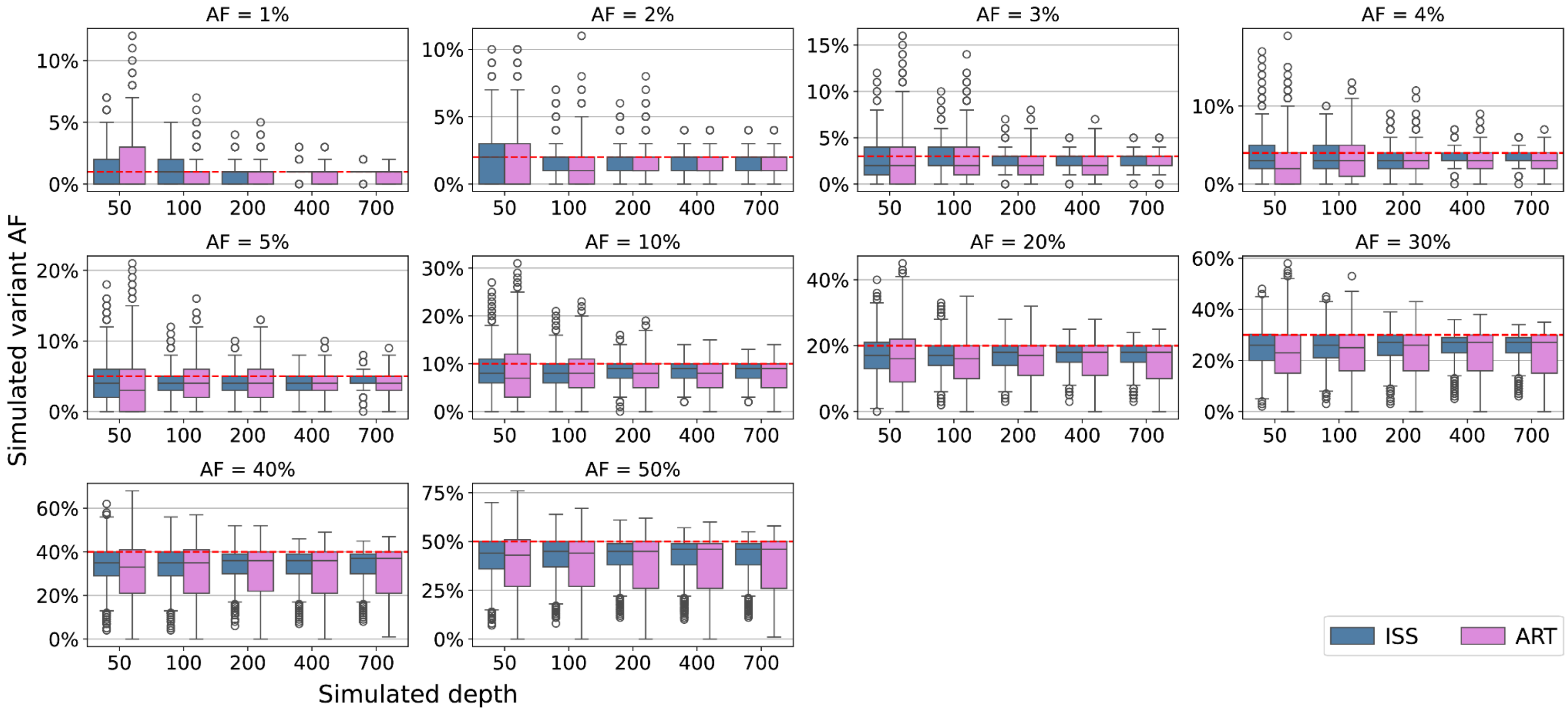

Figure S2 Simulated variant allele frequency achieved by InSilicoSeq (ISS) and ART in each expected sequencing coverage group. The dotted red line indicates the expected allele frequency. The maximum average difference in the deviation of variant allele frequency between depth groups was low at 0.1% for ISS (200x vs 400x, expected AF = 1%) and 0.2% for ART (100x versus 200x, expected AF = 2%).

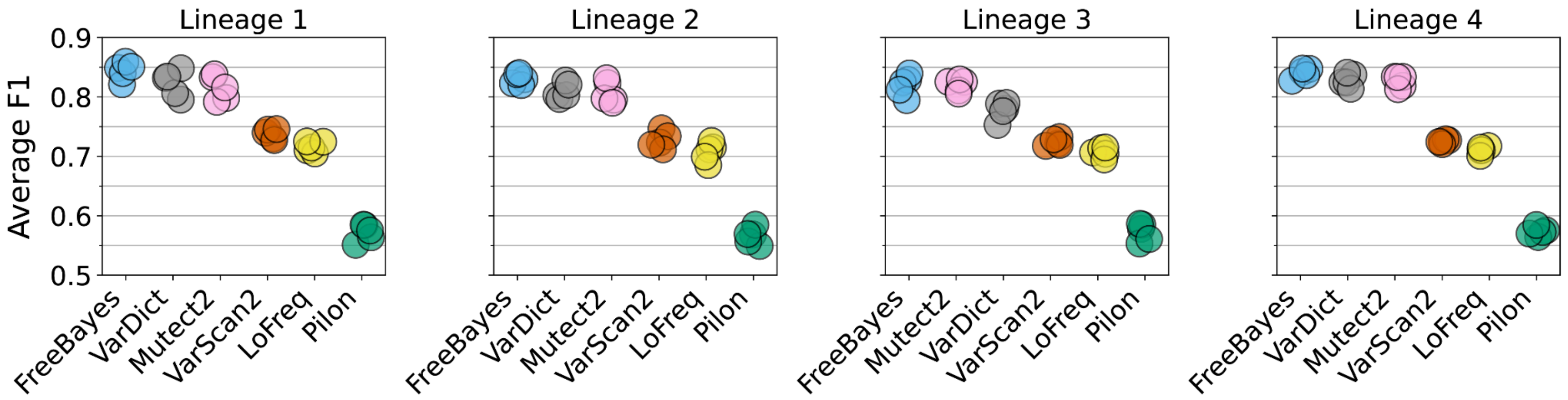

Figure S3 F1 score achieved by each variant caller, averaged over simulated variant AFs and depths. Each point represents the average weighted F1 score for one of the replicate simulations.

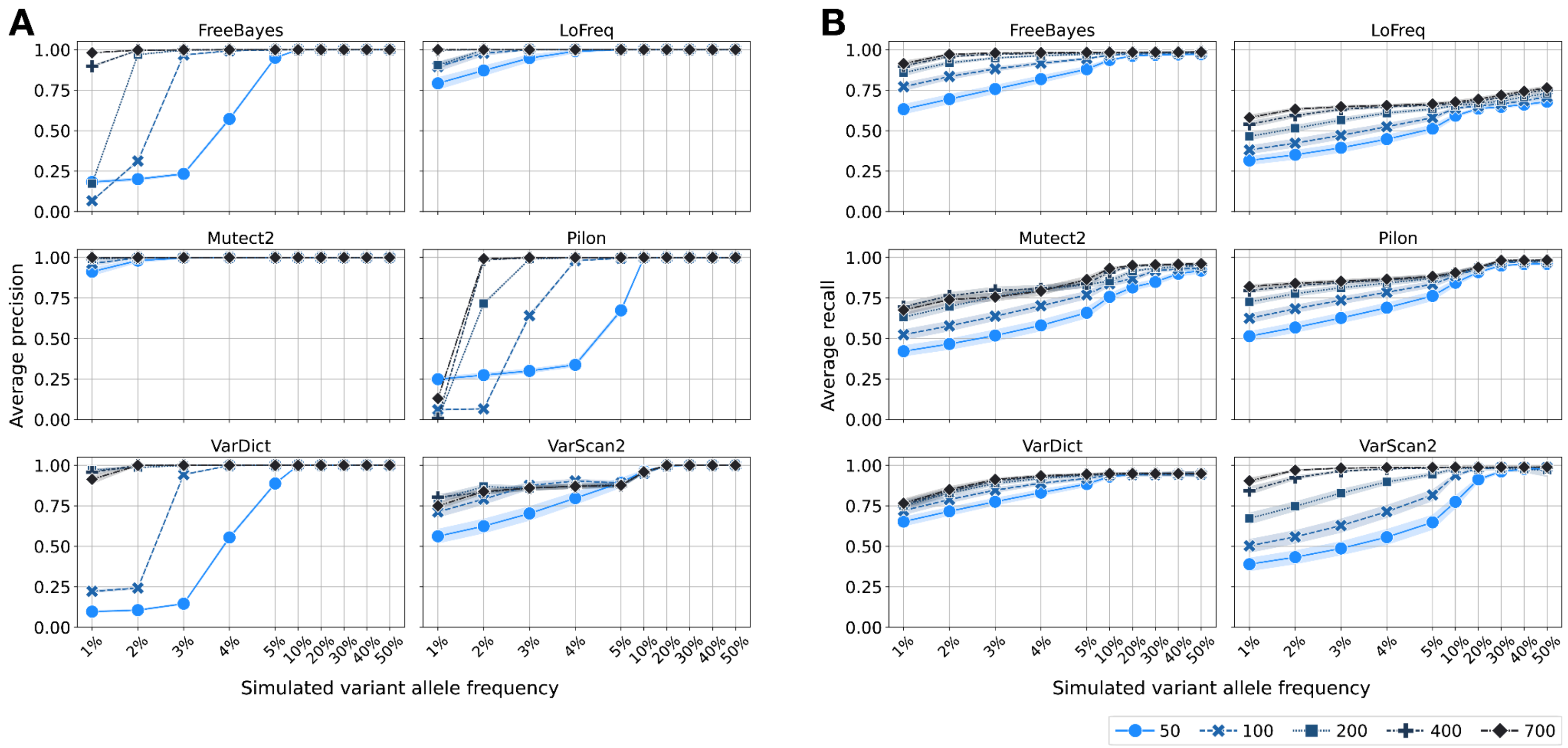

Figure S4 Average precision and recall across variant allele frequency (1-50%) and sequencing depth (50-700x) in the H37Rv samples. **a** Average precision across variant allele frequency and sequencing depth. **b** Average recall across variant allele frequency and sequencing depth. For each tool, the average precision or recall (y-axis) is computed for samples simulated at each sequencing depth, considering variants at some minimum allele frequency (x-axis), averaged over haplotype, depth and replicate. The band around each line represents the 95% confidence interval. Note that the x-axis tick gaps are not proportional to the actual simulated variant AF, and are larger for AF < 10% as this is where the greatest tool-wise differences occur.

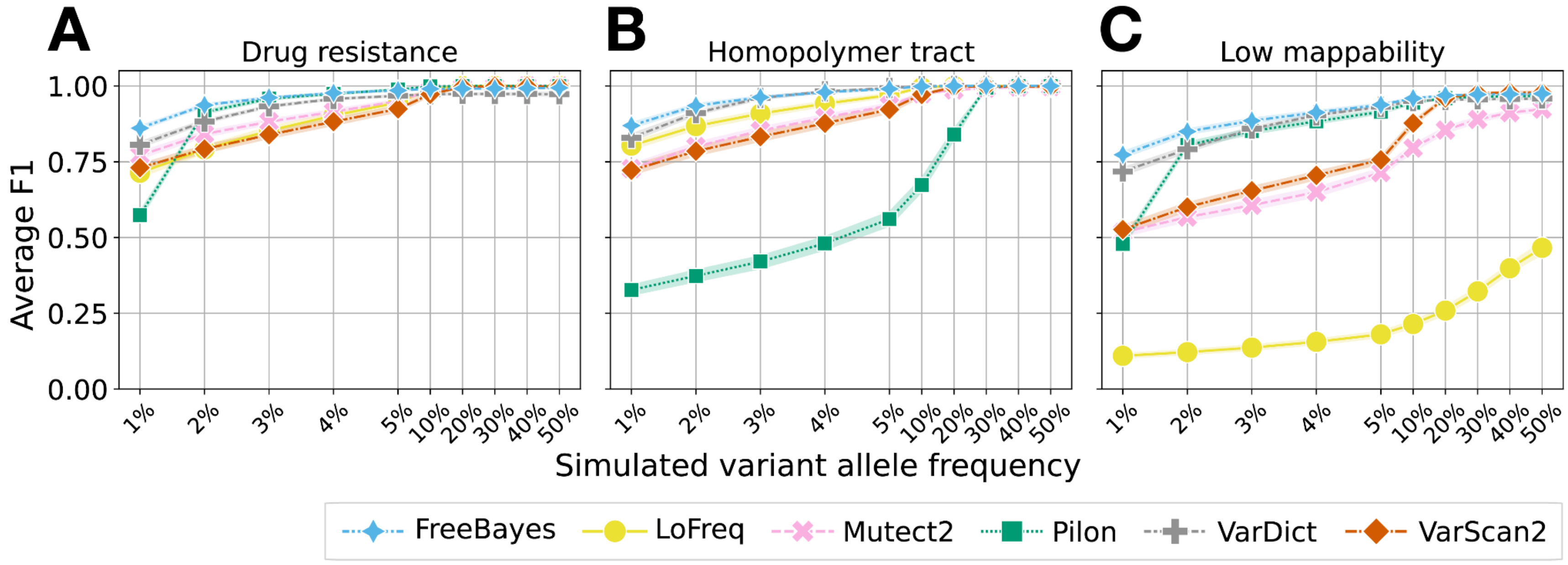

Figure S5 Average cumulative F1 across variant AF pooled over all simulated depths. **a** Average cumulative F1 in drug resistance regions (H37Rv and L1-4 strains). **b** Average cumulative F1 in homopolymer tract regions (H37Rv strains only). **c** Average cumulative F1 in low mappability regions (H37Rv strains only). To compute the average cumulative F1, we computed cumulative precision and recall as a function of increasing minimum variant AF for each of the six tools, averaged over haplotype, depth and replicate. The band around each line represents the 95% confidence interval. Note that the x-axis tick gaps are not proportional to the actual simulated variant AF, and are larger for AF < 10% as this is where the greatest tool-wise differences occur.

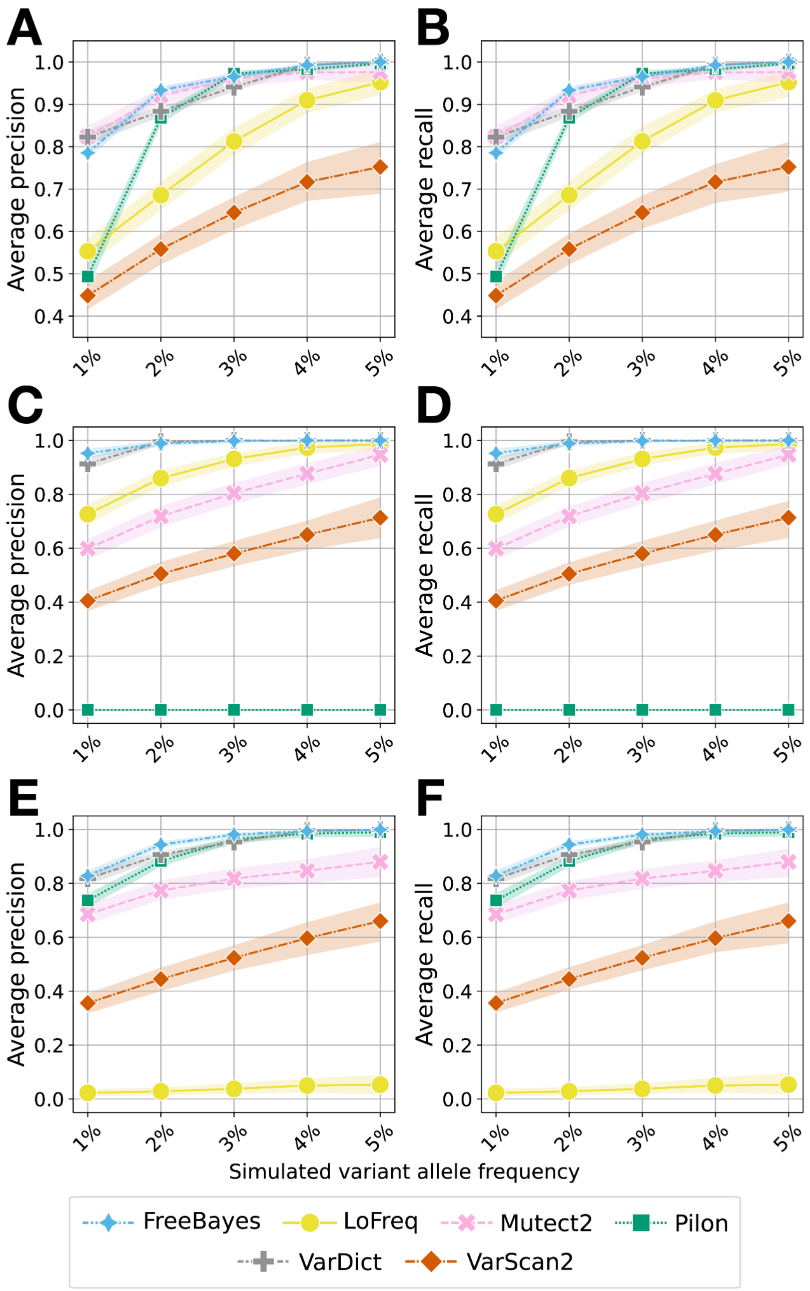

Figure S6 Average cumulative precision and recall across variant AF ≤ 5% pooled over depths of 50x, 100x and 200x. **a, b** Precision and recall in drug resistance (H37Rv and L1-4 strains). **c, d** Precision and recall in homopolymer tract regions. **e, f** Precision and recall in low mappability regions. We computed cumulative precision and recall as a function of increasing minimum variant AF for each of the six tools, averaged over haplotype, depths 50-200x and replicate, considering only variants detected at AF ≤ 5%. The band around each line represents the 95% confidence interval. Note that the y-axis is from 0.4-1.0 for A and B, and 0-1.0 for C-F. The x-axis tick gaps are not proportional to the actual simulated variant AF, and are larger for AF < 10% as this is where the greatest tool-wise differences occur.

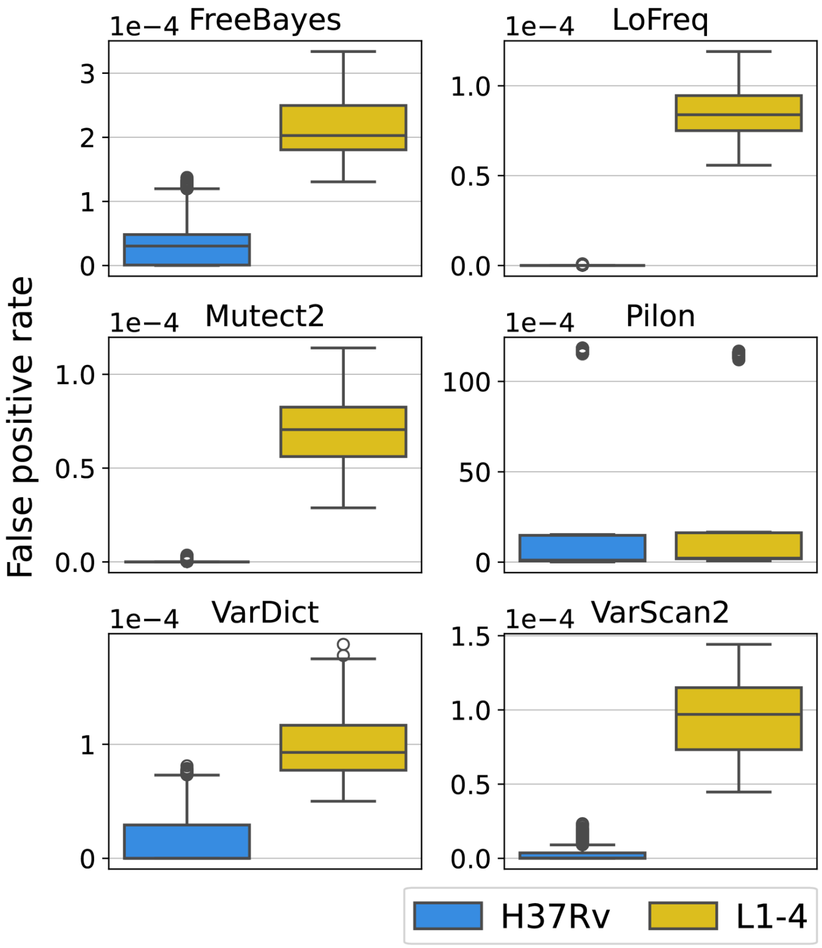

Figure S7 Genome-wide false positive rate in H37Rv versus L1-4 strains for AF 5-50%. False positive rate is calculated per-base across the entire genome. The average genome-wide FPRs for the FreeBayes, LoFreq, Mutect2, Pilon, VarDict and VarScan2 are 3.96E-05, 4.81E-08, 3.63E-08, 2.67E-03, 1.91E-05 and 2.43E-06 respectively for the H37Rv strains, and 2.15E-04, 8.5E-05, 6.9E-05, 2.71E-03, 9.7E-05 and 9.5E-05 respectively for the L1-4 strains. The pairwise comparisons for each tool are statistically significant after Benjamini-Hochberg correction (Mann-Whitney U test with FDR = 0.05).

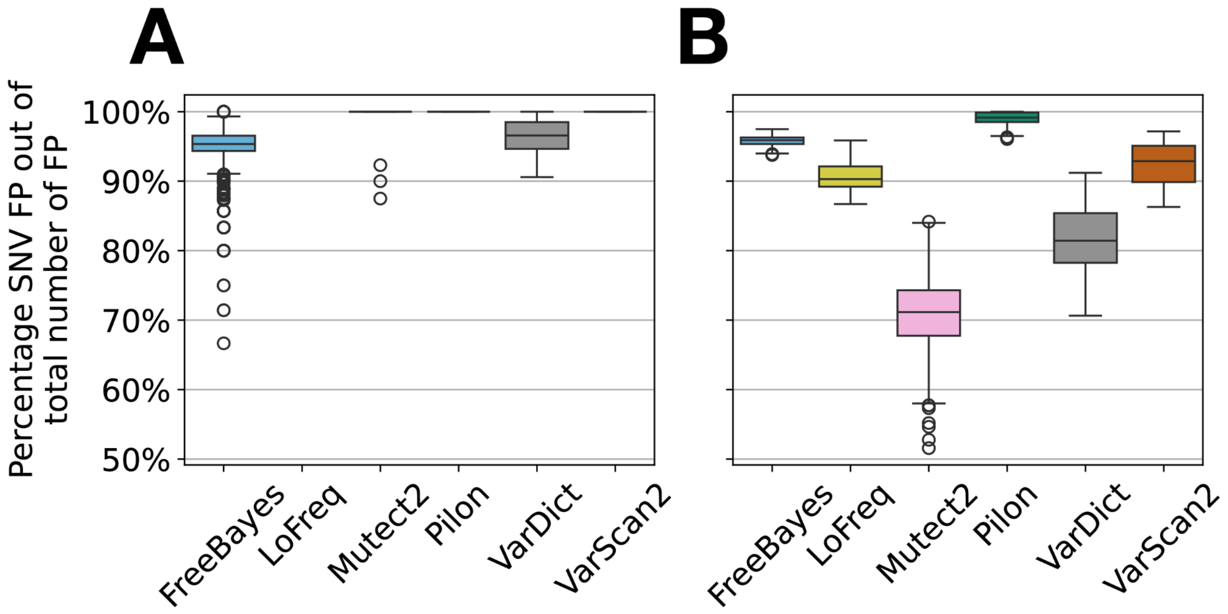

Figure S8 Distribution of the percentage of FPs that are SNVs per tool and genome background. **a** The distribution of SNV FP percentages per strain in H37Rv strains. **b** The distribution of SNV FP percentages per strain in L1-4 strains. For each tool, the percentage of FPs detected in a single strain that are SNVs was calculated for all strains with at least 5 FPs detected by that tool.

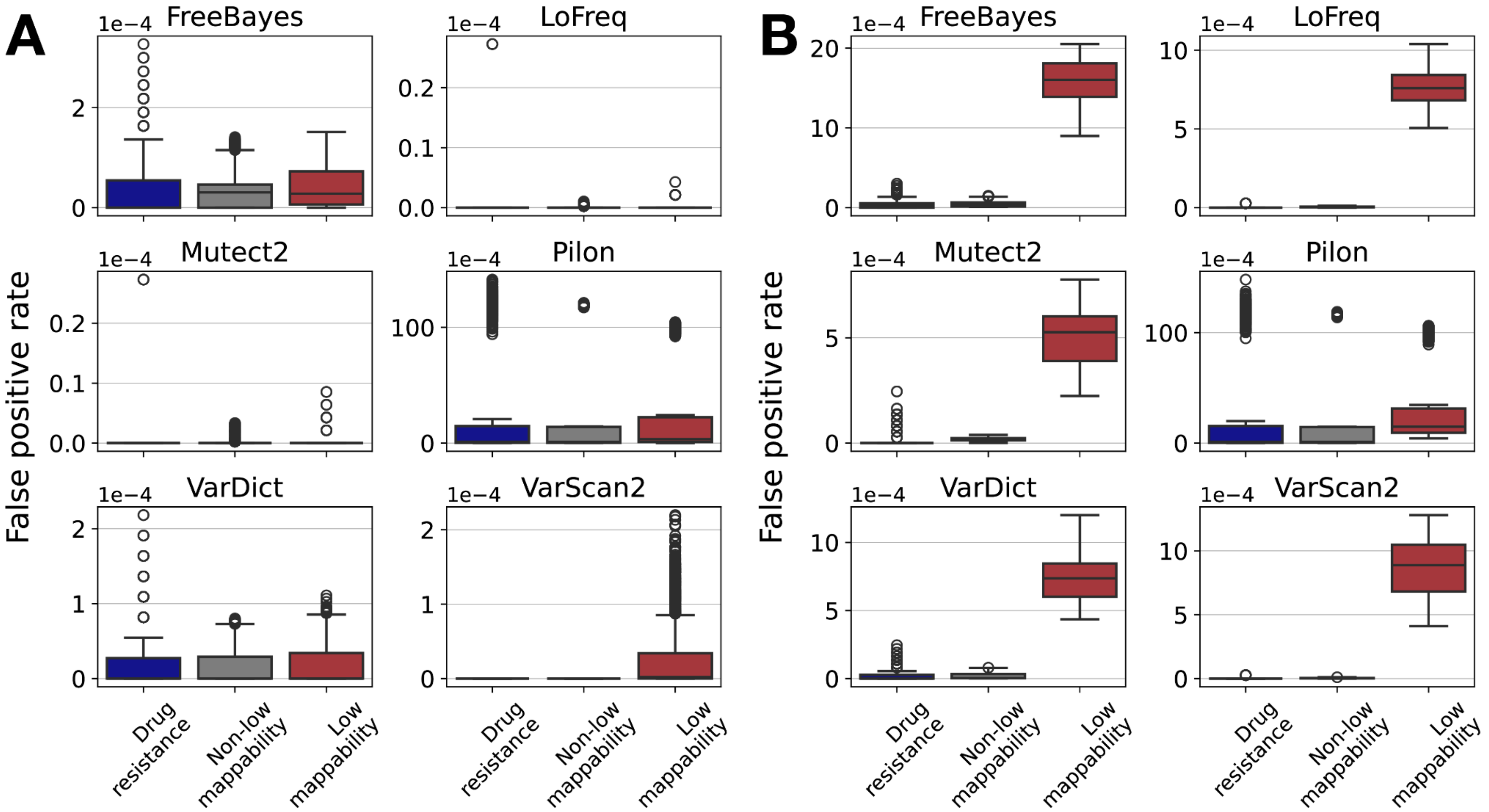

Figure S9 False positive rates by tool in drug resistance, non-low mappability and low mappability regions. **a** The distribution of false positive rates per strain and region in H37Rv strains. **b** The distribution of false positive rates per strain and region in L1-4 strains. Each region is defined to be mutually exclusive for this comparison i.e. the non-low mappability regions do not include the drug resistance regions. Each box plot shows the distribution of FPRs in each region and for each tool. Note that the subplots do not share the same y-axis. The pairwise comparisons between each region for each tool are statistically significant in both groups of strains after Benjamini-Hochberg correction (Mann-Whitney U test with FDR = 0.05).

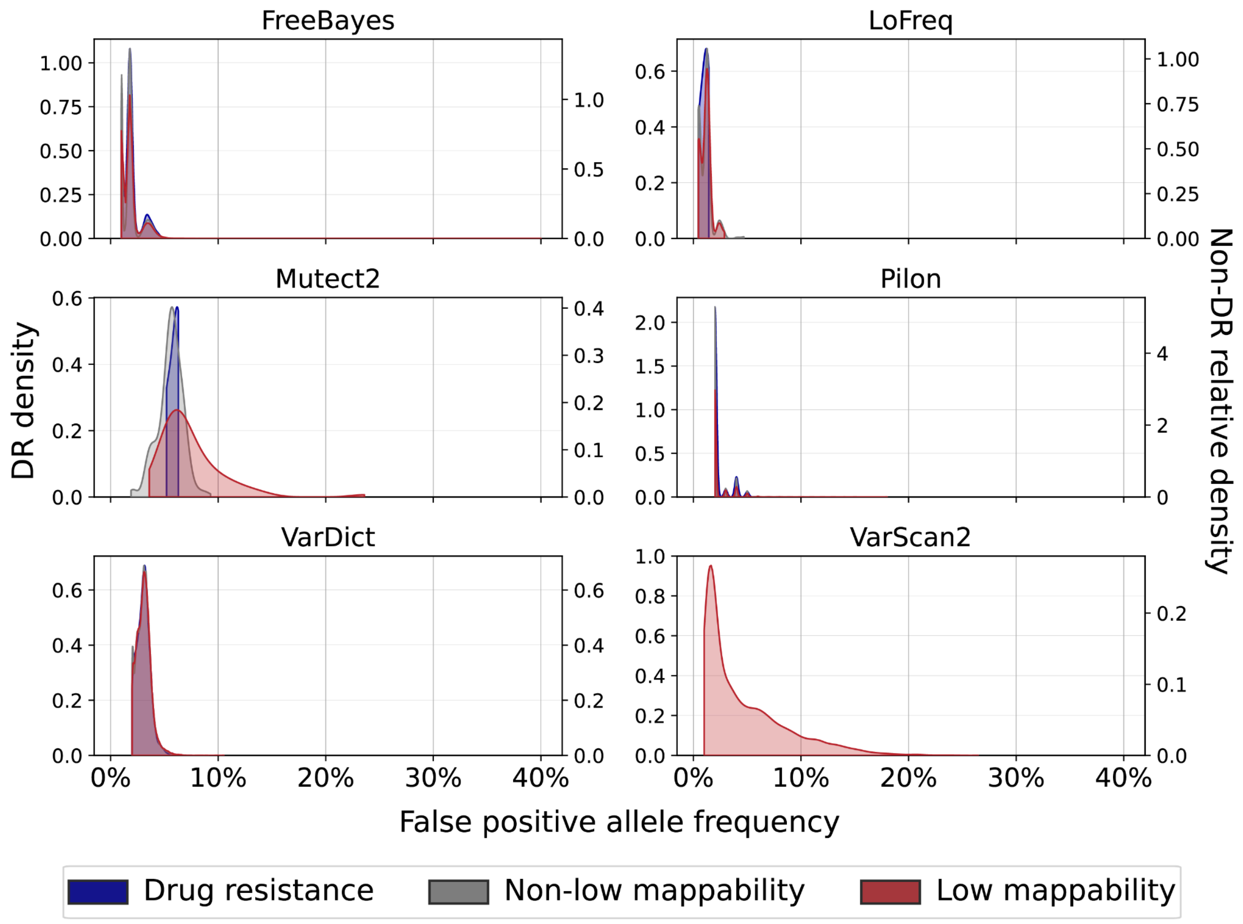

Figure S10 False positive allele frequency distribution in the H37Rv strains by region. Each region is defined to be mutually exclusive for this comparison i.e. the non-low mappability regions do not include the drug resistance regions. The left y-axis displays the densities of the DR AF distributions, and the right y-axis displays the relative densities of the low mappability (non-DR) and non-low mappability (non-DR) AF distributions (normalized independently). For Pilon we include only the FP with AF > 1% (a median of 99.96% of Pilon FPs across all strains have AF = 1%). All DR FP and non-low mappability FP occur at AF < 7% and AF ≤ 10% respectively. FP in low mappability regions occur at AFs 1-40%.

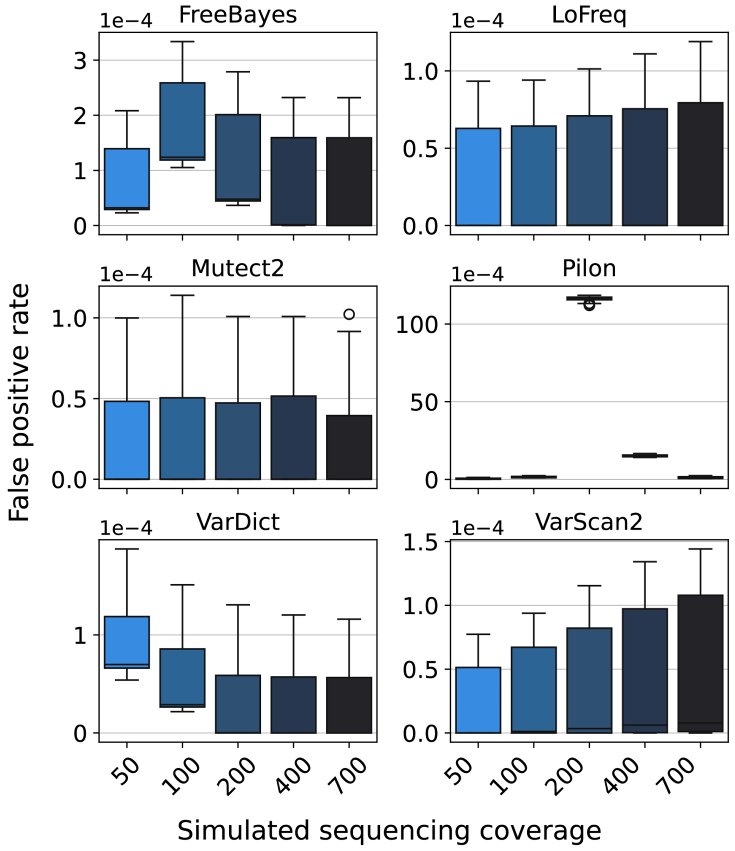

Figure S11 Genome-wide false positive rate across all strains in each simulated sequencing coverage group. False positive rate is calculated per-base across the entire genome. All tools were run on the same simulated strains, and further, the excessively high number of FP detected by Pilon in strains simulated at 200x is consistent with the excess number of variants detected by Pilon in clinical isolates with depths ~ 200x (see Supplementary Results D), motivating a lack of simulation bias in this result.

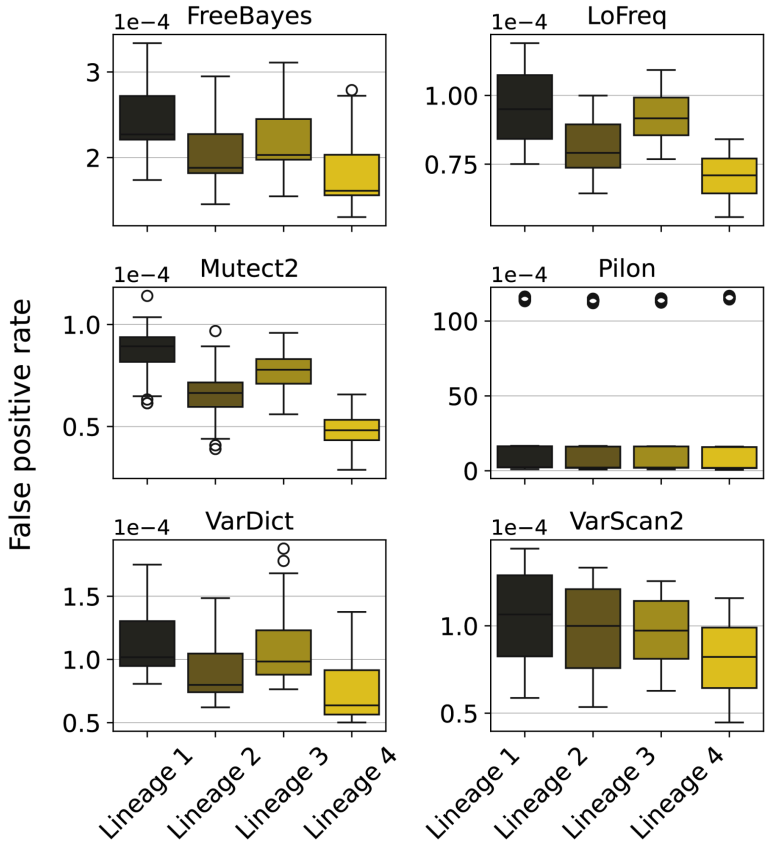

Figure S12 Genome-wide false positive rate by lineage background (lineages 1-4). False positive rate is calculated per-base across the entire genome. The largest difference between any one pair of lineages for any tool occurs between the FPRs for lineages 1 and 4 in FreeBayes (average FPR is 2.5E-04 and 1.8E-04 respectively).

Figures S13-17 Distributions of read mapping and quality characteristics at variant sites split by true and false positives in the L1-4 strains for each tool.

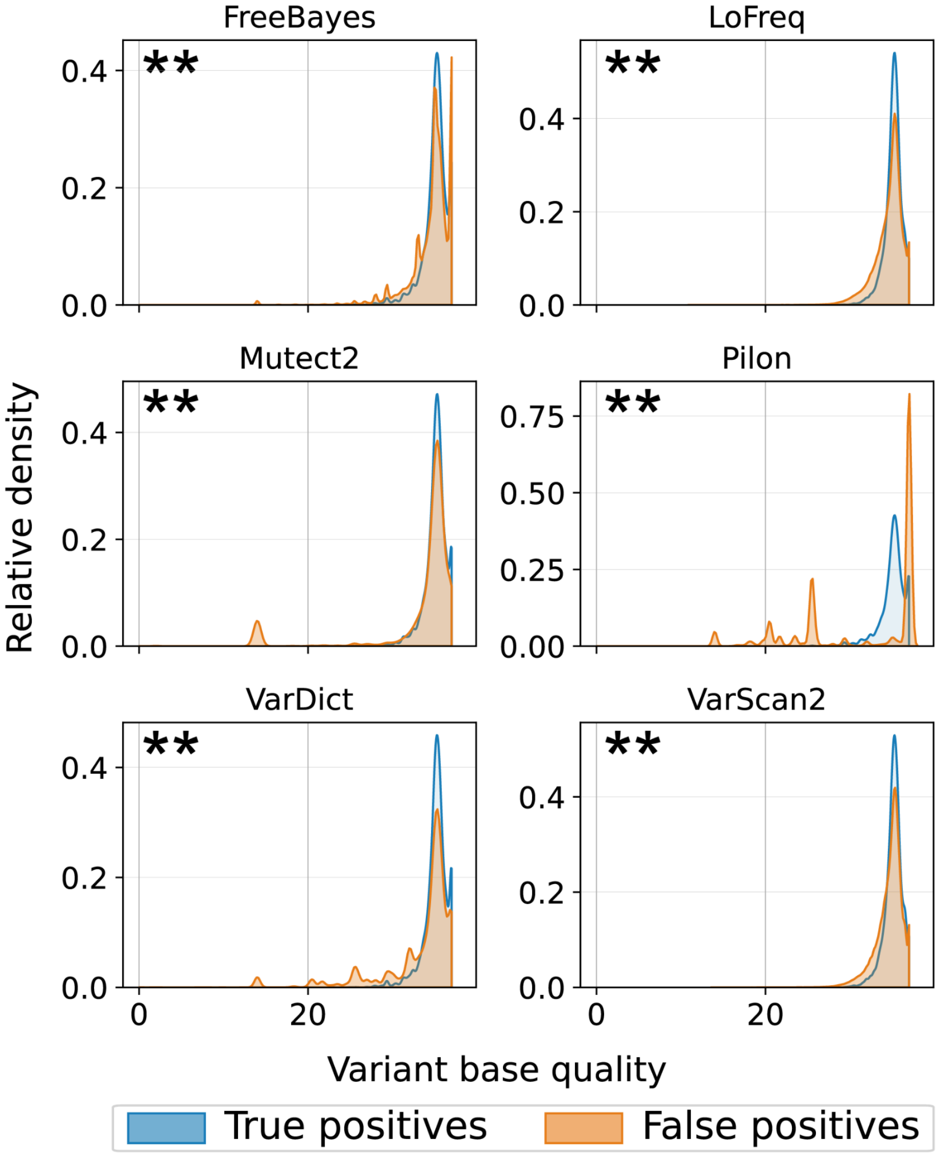

Figure S13 Variant allele base quality distribution at true and false positive variant sites. The differences between the TP and FP distributions are statistically significant for all tools after Benjamini-Hochberg correction (indicated by asterisks; Mann-Whitney U test with FDR = 0.05).

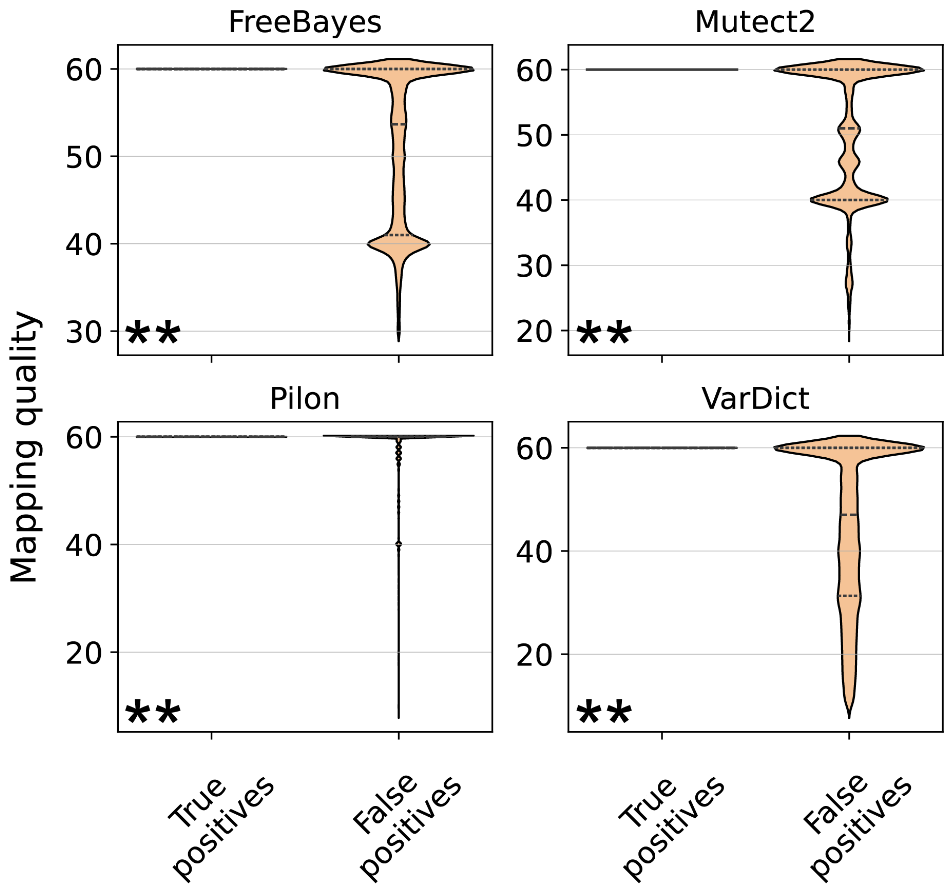

Figure S14 Mapping quality distribution at true and false positive variant sites. Mapping quality was available for variant calls made by FreeBayes, Mutect2, Pilon and VarDict only. The differences between the TP and FP distributions are statistically significant for all tools after Benjamini-Hochberg correction (indicated by asterisks; Mann-Whitney U test with FDR = 0.05).

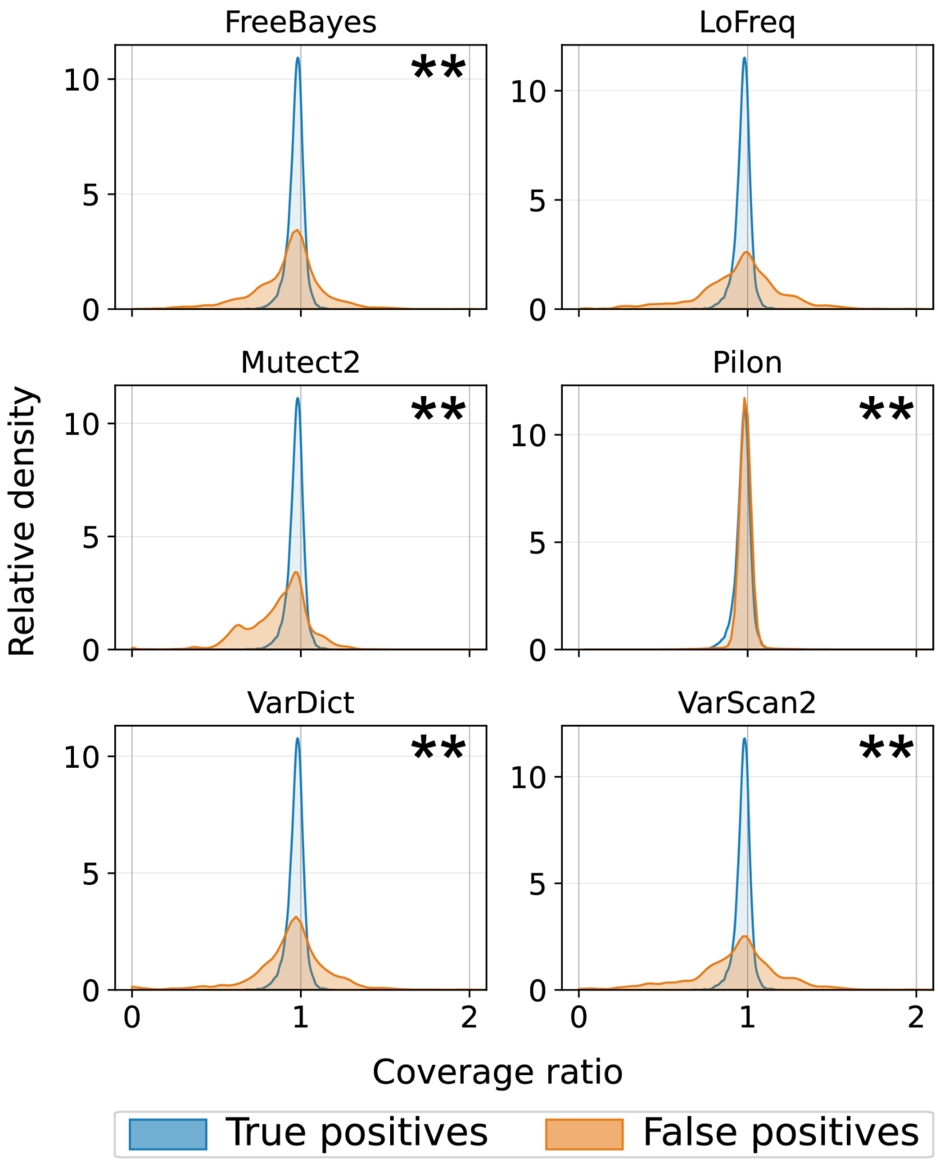

Figure S15 Coverage ratio distribution at true and false positive variant sites. The coverage ratio is equal to the coverage at a variant site relative to the average regional coverage. The x-axis is cut-off at 2. Across all tools 0% of TP sites and 0.01-0.22% of FP sites have a coverage ratio > 2. The differences between the TP and FP distributions are statistically significant for all tools except LoFreq after Benjamini-Hochberg correction (indicated by asterisks; Mann-Whitney U test with FDR = 0.05).

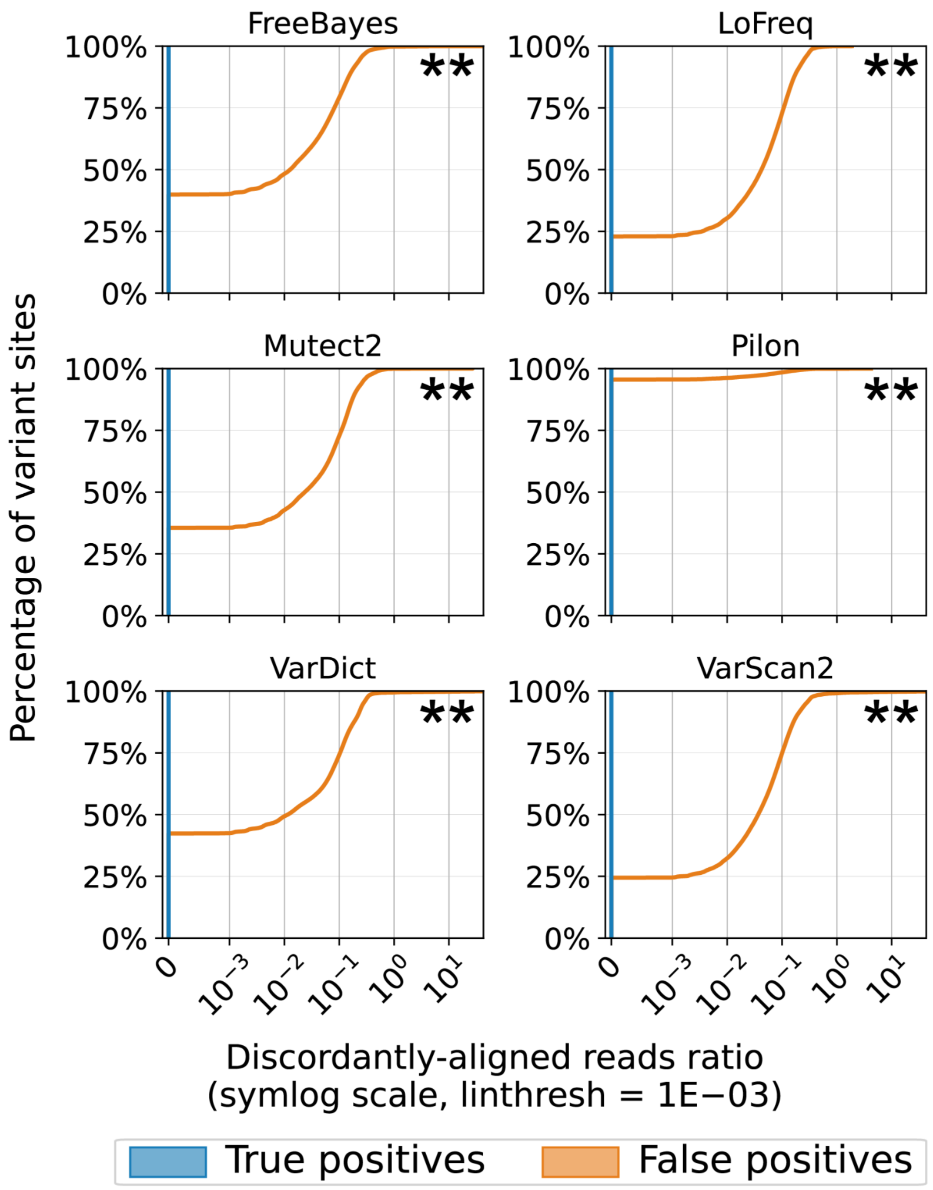

Figure S16 Discordantly-aligned reads ratio distribution at true and false positive variant sites. The discordantly-aligned read ratio is equal to the number of discordantly-aligned reads at a variant site relative to site coverage. The empirical cumulative distribution function is shown on a symmetric logarithmic x-axis with a linear threshold of 1E-03. Across all tools 100% of TP sites and 23-96% of FP sites have a discordantly-aligned reads ratio of 0. The ratio distributions for Pilon are the least practically different between TPs and FPs. The differences between the TP and FP distributions are statistically significant for all tools after Benjamini-Hochberg correction (indicated by asterisks; Mann-Whitney U test with FDR = 0.05).

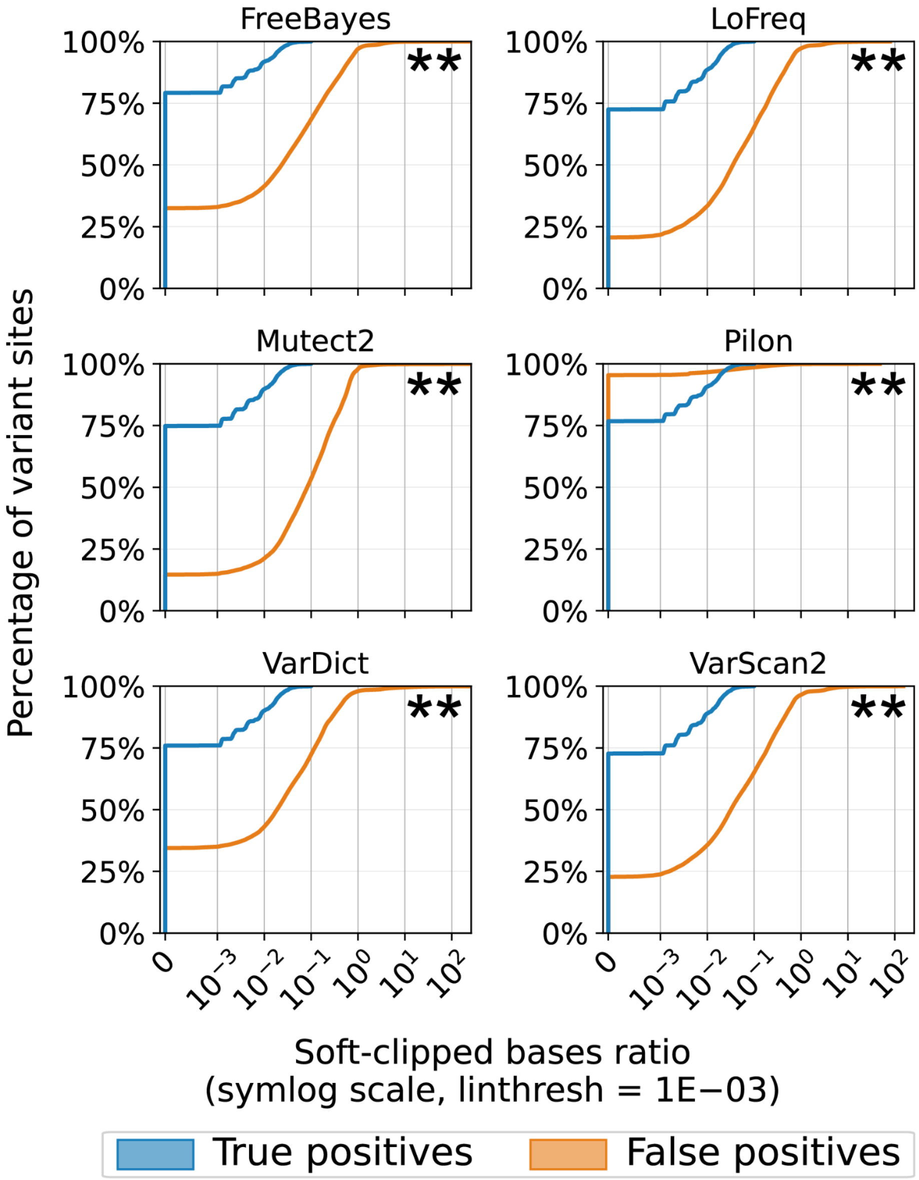

Figure S17 Soft-clipped bases ratio distribution at true and false positive variant sites. The soft-clipped bases ratio is equal to the number of soft-clipped bases at a variant site relative to site coverage. The empirical cumulative distribution function is shown on a symmetric logarithmic x-axis with a linear threshold of 1e-3. Across all tools 72-79% of TP sites and 15-95% of FP sites have a soft-clipped bases ratio of 0. Pilon is the only tool to exhibit a higher percentage of false positive calls at variant sites with a soft-clipped bases ratio of 0, which is likely due to the fact that a median of 42% of the FPs called by Pilon per L1-4 strain occur at AF = 1%. The differences between the TP and FP distributions are statistically significant for all tools after Benjamini-Hochberg correction (indicated by asterisks; Mann-Whitney U test with FDR = 0.05).

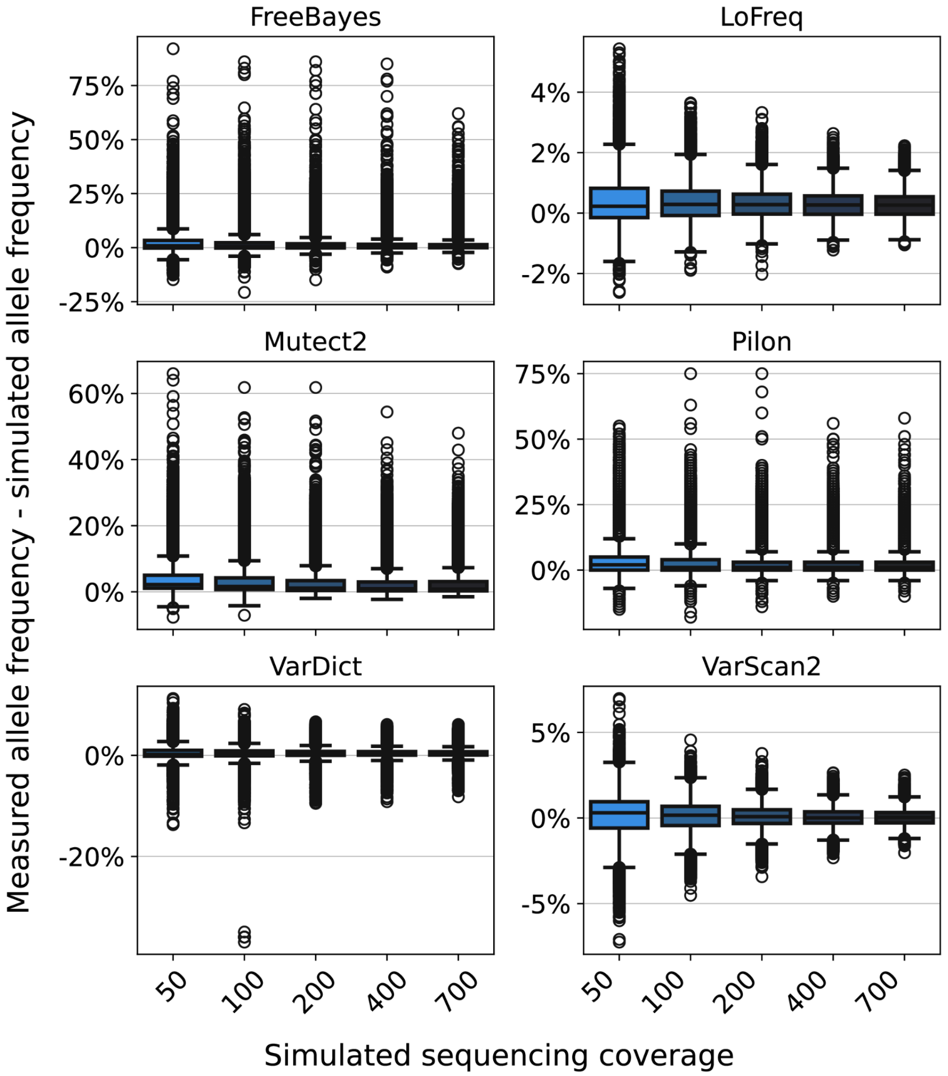

Figure S18 Distribution of the difference between the measured allele frequency and the simulated allele frequency for variants simulated at each sequencing depth. While the differences between some pairwise depth comparisons are statistically significant after Benjamini-Hochberg correction (Mann-Whitney U test with FDR = 0.05), the maximum average difference of the measured AF and simulated AF for any pair of depths and any tool is -1.57%. Note that the subplots do not share the same y-axis.

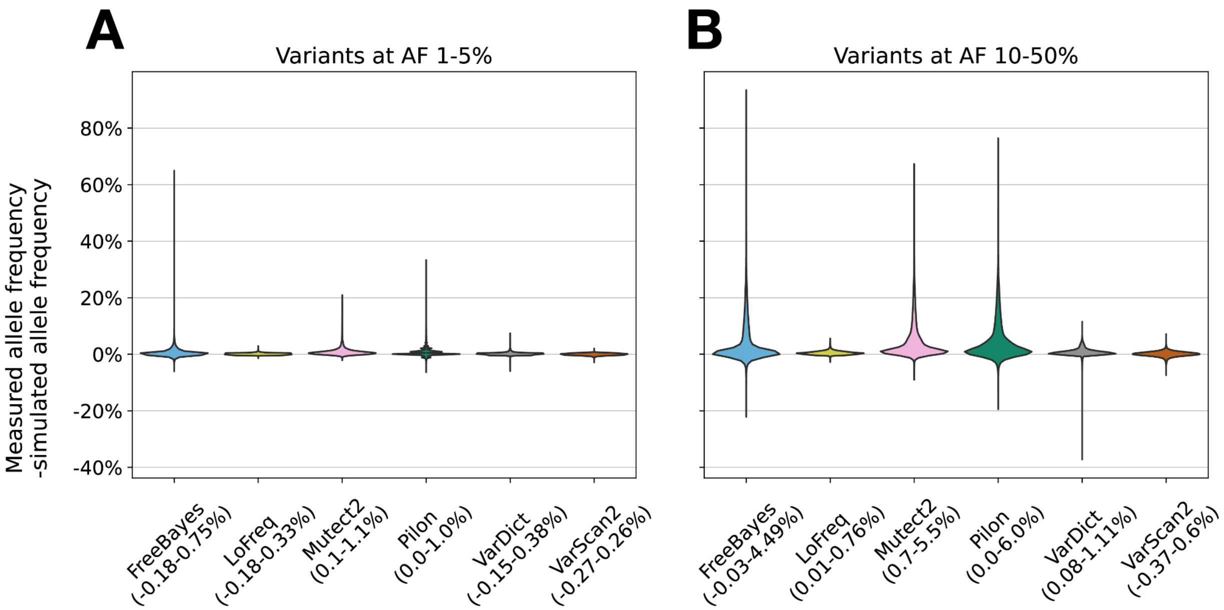

Figure S19 Distribution of the difference between the measured and simulated allele frequency for variants across all variants, depths, haplotypes and replicates. **a** Distribution for variants simulated at 1% ≤ AF ≤ 5%. **b** Distribution for variants simulated at 10% ≤ AF ≤ 50%. The IQR for each distribution is displayed next to the tool name. Overall, as expected, the highest AF differences are observed for variants simulated at an AF in the higher range (10-50%).

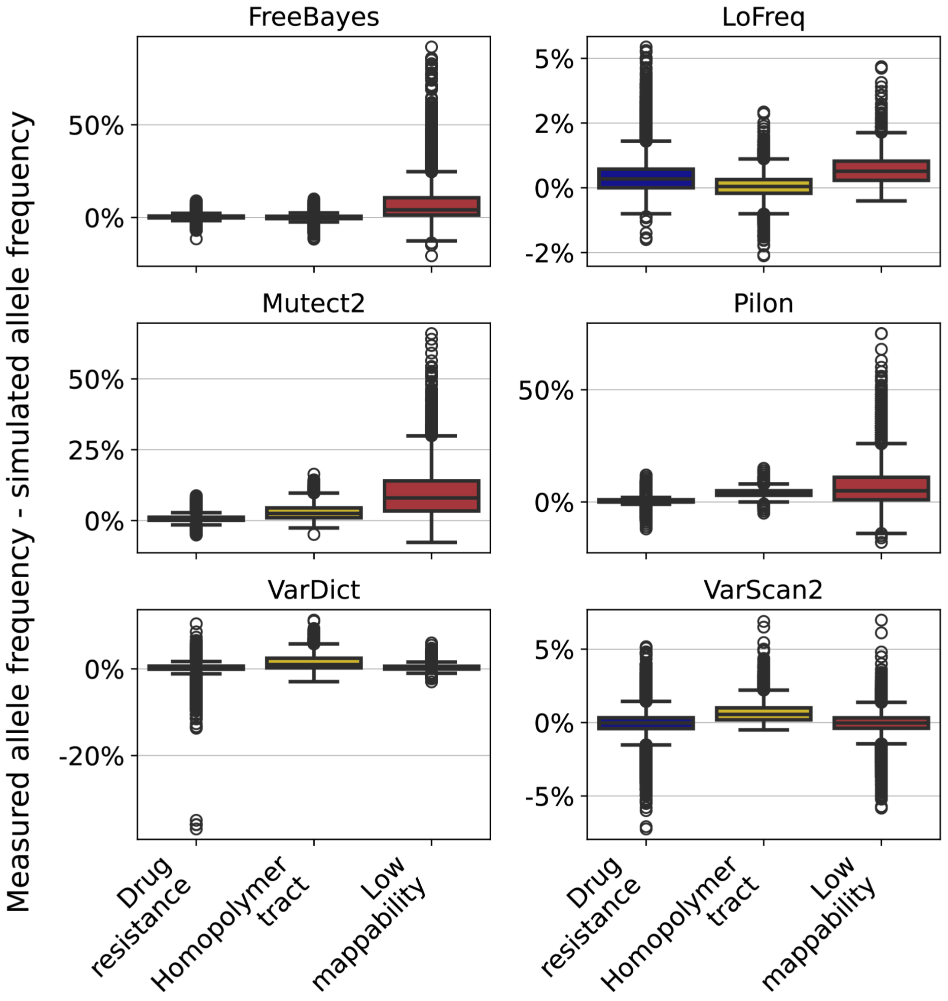

Figure S20 Distribution of the difference between the measured allele frequency and the simulated allele frequency for variants simulated in each mutation region. All pairwise comparisons are statistically significant after Benjamini-Hochberg correction (Mann-Whitney U test with FDR = 0.05), except the DR-LM comparisons for VarDict and VarScan2. The average difference in measured and simulated allele frequency across all tools, however, is not substantially different between DR and HT regions for all tools except Mutect2 and Pilon (mean AF difference for Mutect2 and Pilon: DR = 0.58%, HT = 3.33%; mean AF difference for other tools: DR = 0.24%, HT = 0.54%). The AFs of LM variants measured by FreeBayes, Mutect2 and Pilon are practically the most different from those in any other region measured by these tools, or those measured in all other regions by any other tool (mean AF difference: FreeBayes, Mutect2 and Pilon LM = 7.91%; FreeBayes, Mutect2 and Pilon DR and HT = 1.36%, LoFreq, VarDict and VarScan2 all regions = 0.42%). Note that the subplots do not share the same y-axis.

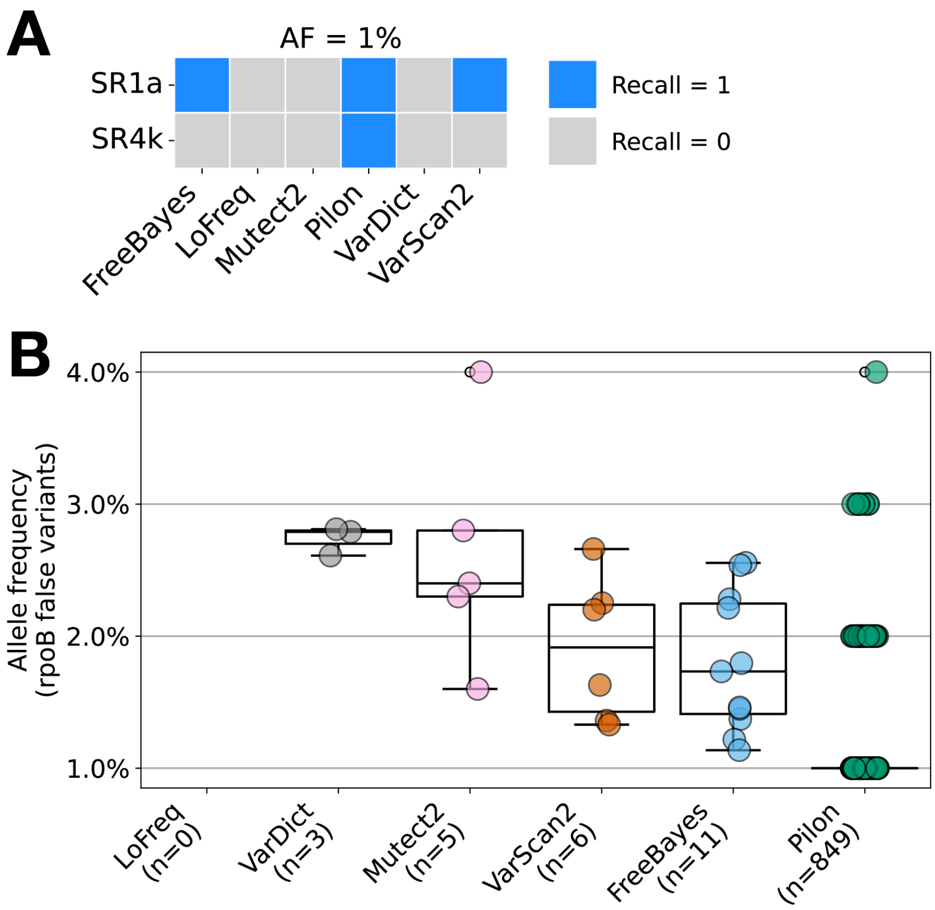

Figure S21 Variant caller performance in in-vitro samples with introduced *rpoB* mutations across all allele frequencies (*rpoB* only). **a** Heatmap of recall for each tool on each in-vitro isolate with mutations introduced at AF = 1%. A Ser531Leu mutation was introduced in the SR1a isolates; a His526Pro mutation was introduced in the SR4k isolates. **b** Distribution of the false variants AFs detected in *rpoB* by each tool. The number of total false variants detected by a tool is shown in parentheses below each tool name.

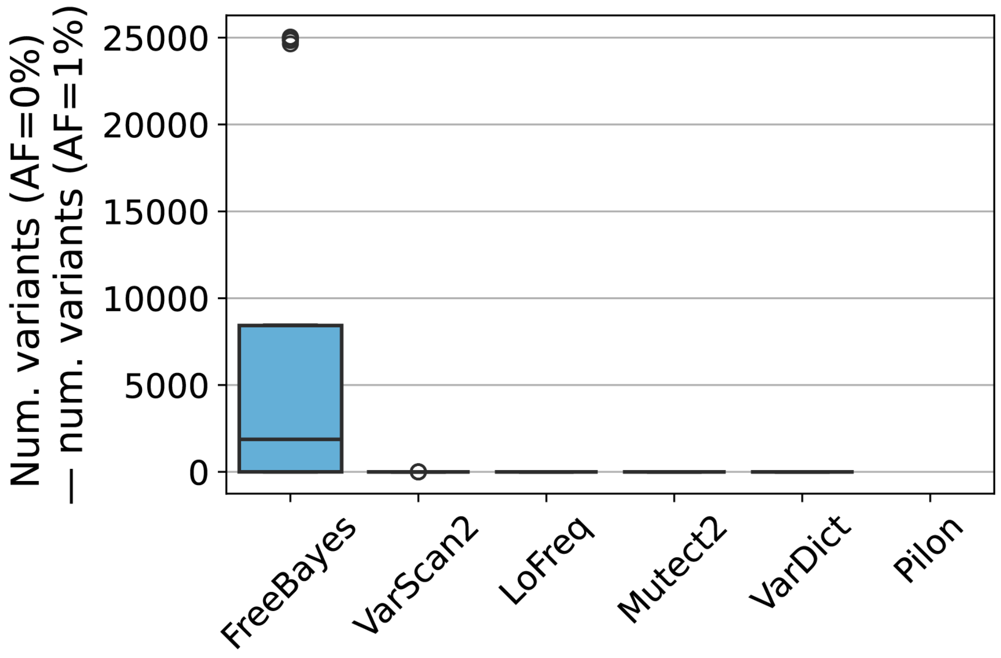

Figure S22 Distribution of the difference in the number of variants called by each tool (except Pilon) when the minimum variant allele frequency is set to 0% versus 1%. FreeBayes called, on average, 7018 more variants. VarScan2 called at most 5 more variants per strain. No difference was observed in the number of variants called by LoFreq, Mutect2 and VarDict. Pilon was excluded because 1% is the minimum reported quality-weighted allele frequency for a call.

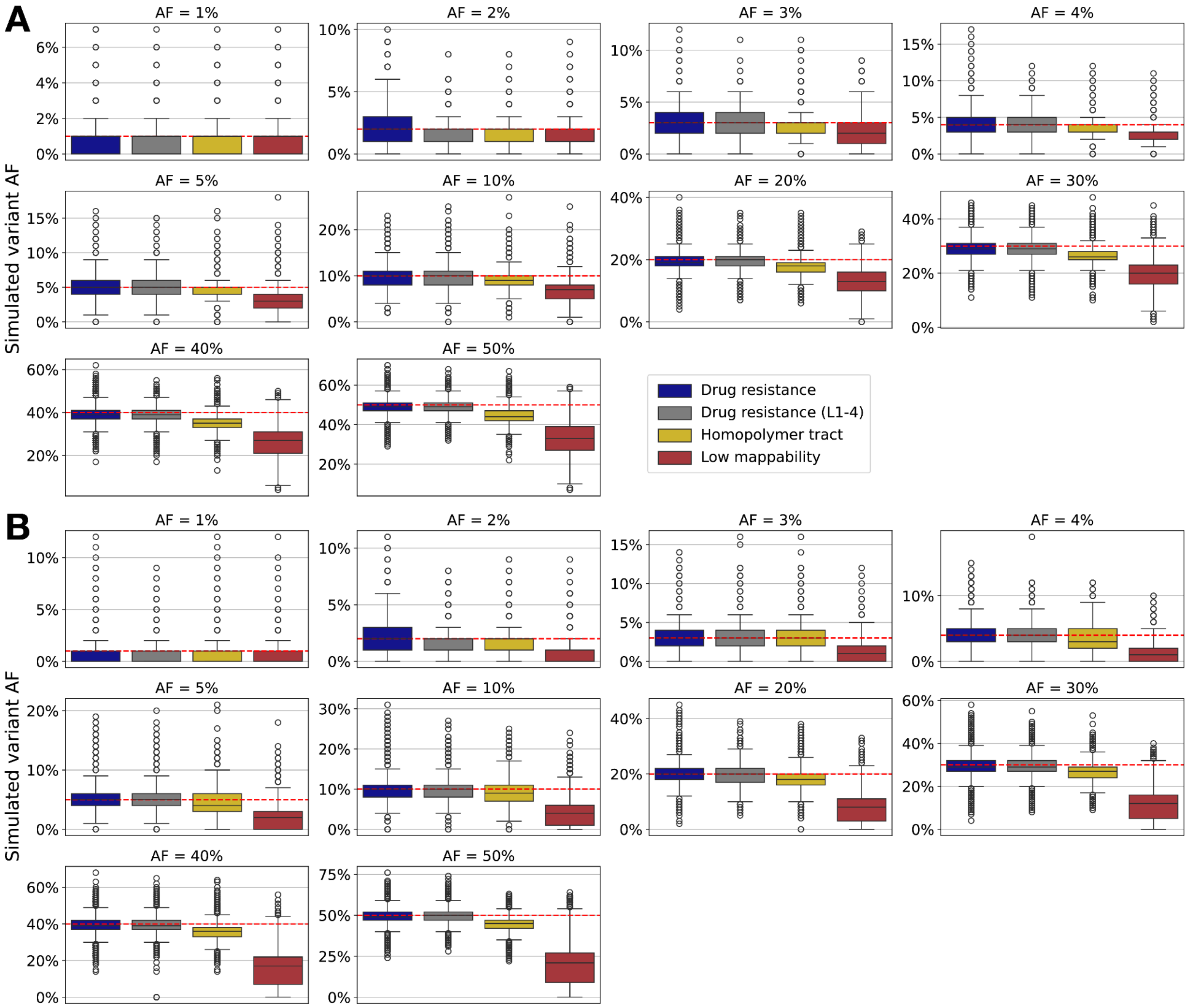

Figure S23 Simulated variant allele frequency achieved in each genomic region considered. **a** Simulated AFs achieved by InSilicoSeq (ISS). **b** Simulated AFs achieved by ART. The red dotted line indicates the expected allele frequency. The simulated variant AFs tend to be lower than expected in each region, and this is most pronounced in LM regions.

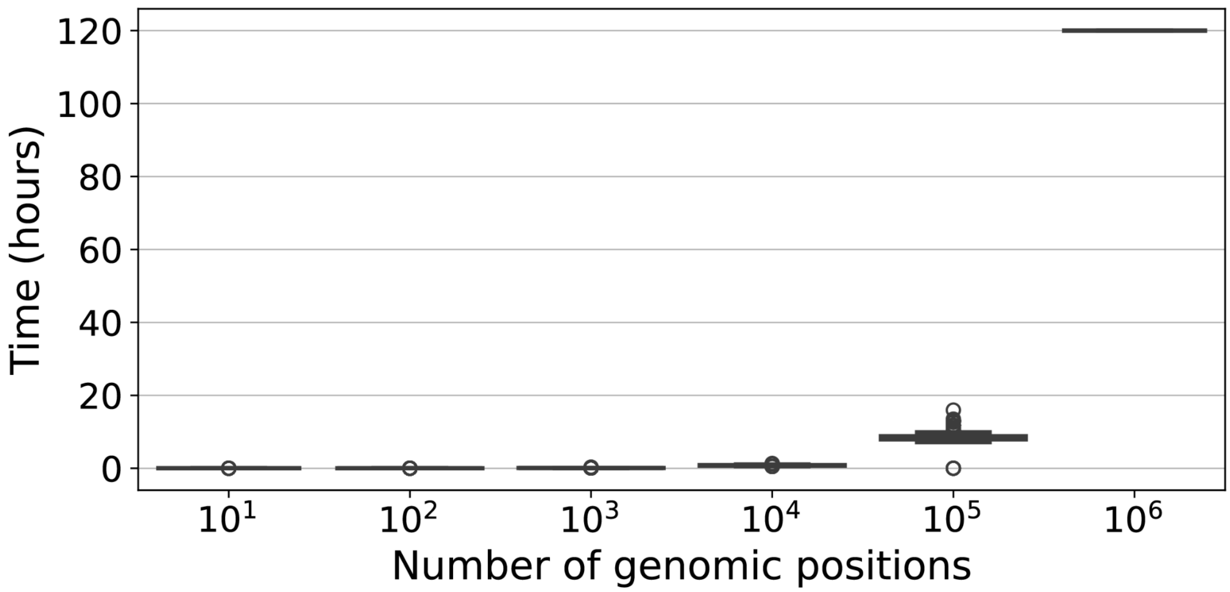

Figure S24 BinoSNP CPU runtime for a range of input positions from 10^1-10^6. The distribution of the runtimes for each input position set is shown for 500 strains. The BinoSNP runs with 10^6 positions timed out after 5 days.

Figures S25-27 Simulated mutations in the L1-4 strains consistently missed by FreeBayes and VarDict.

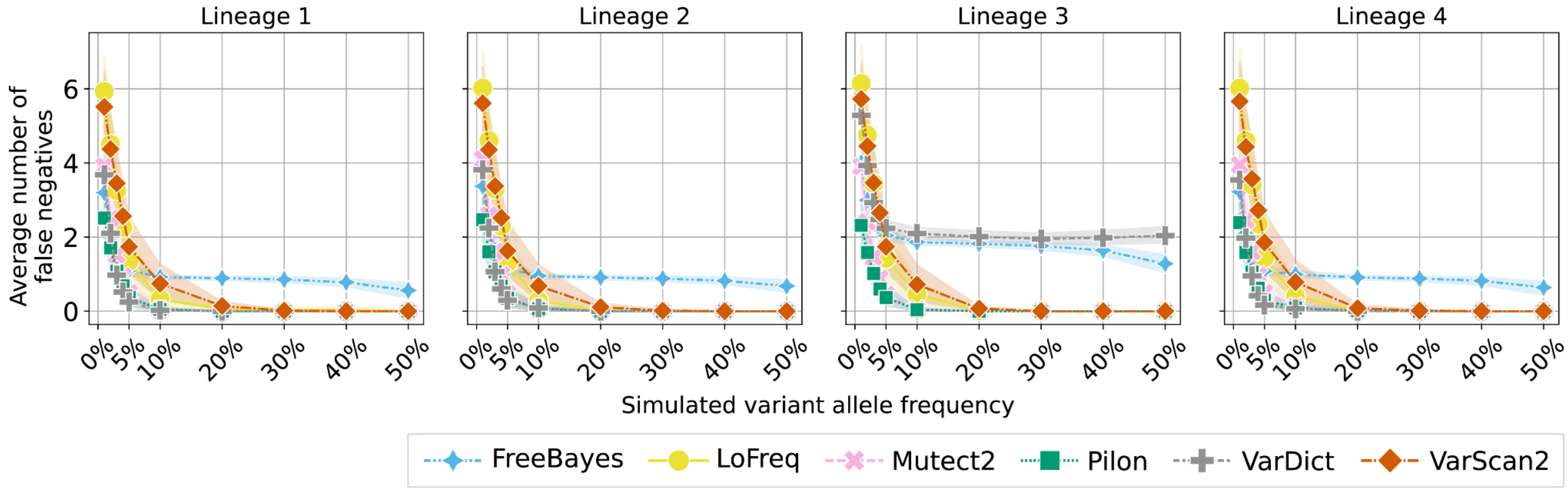

Figure S25 Average number of false negatives for each tool across variant AFs for mutations introduced in DR regions only (L1-4 strains).

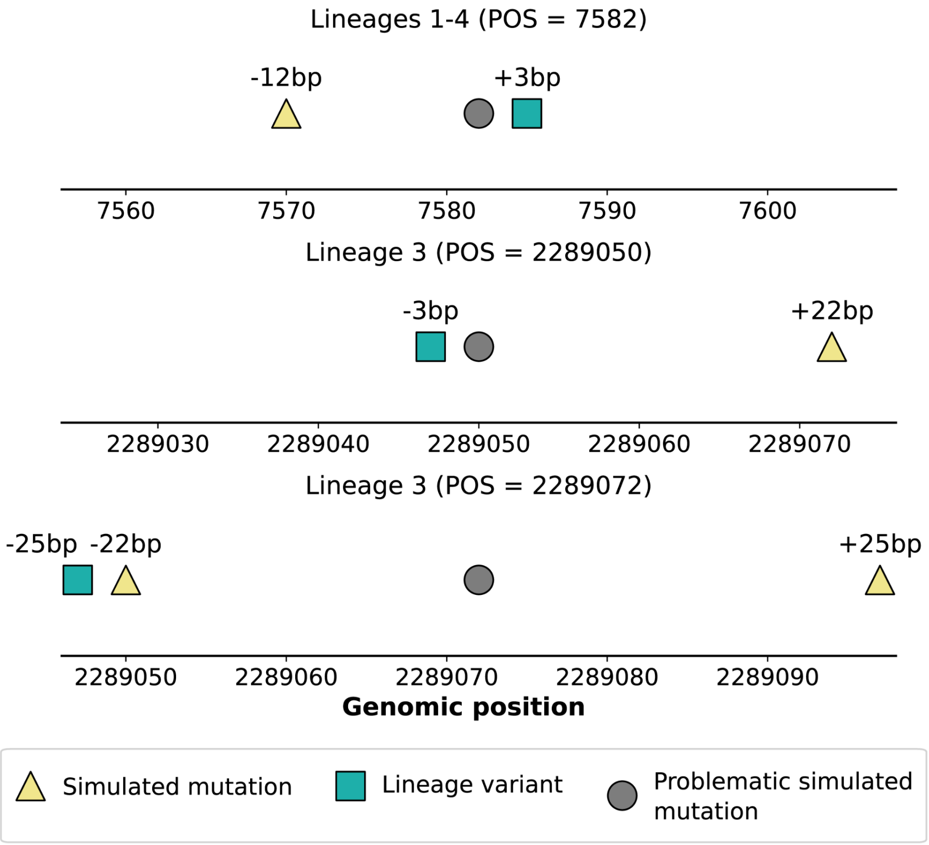

Figure S26 Distance between problematic simulated variants at DR positions and nearby (within 25bp) (1) nearby lineage variants, and (2) additional simulated variants.

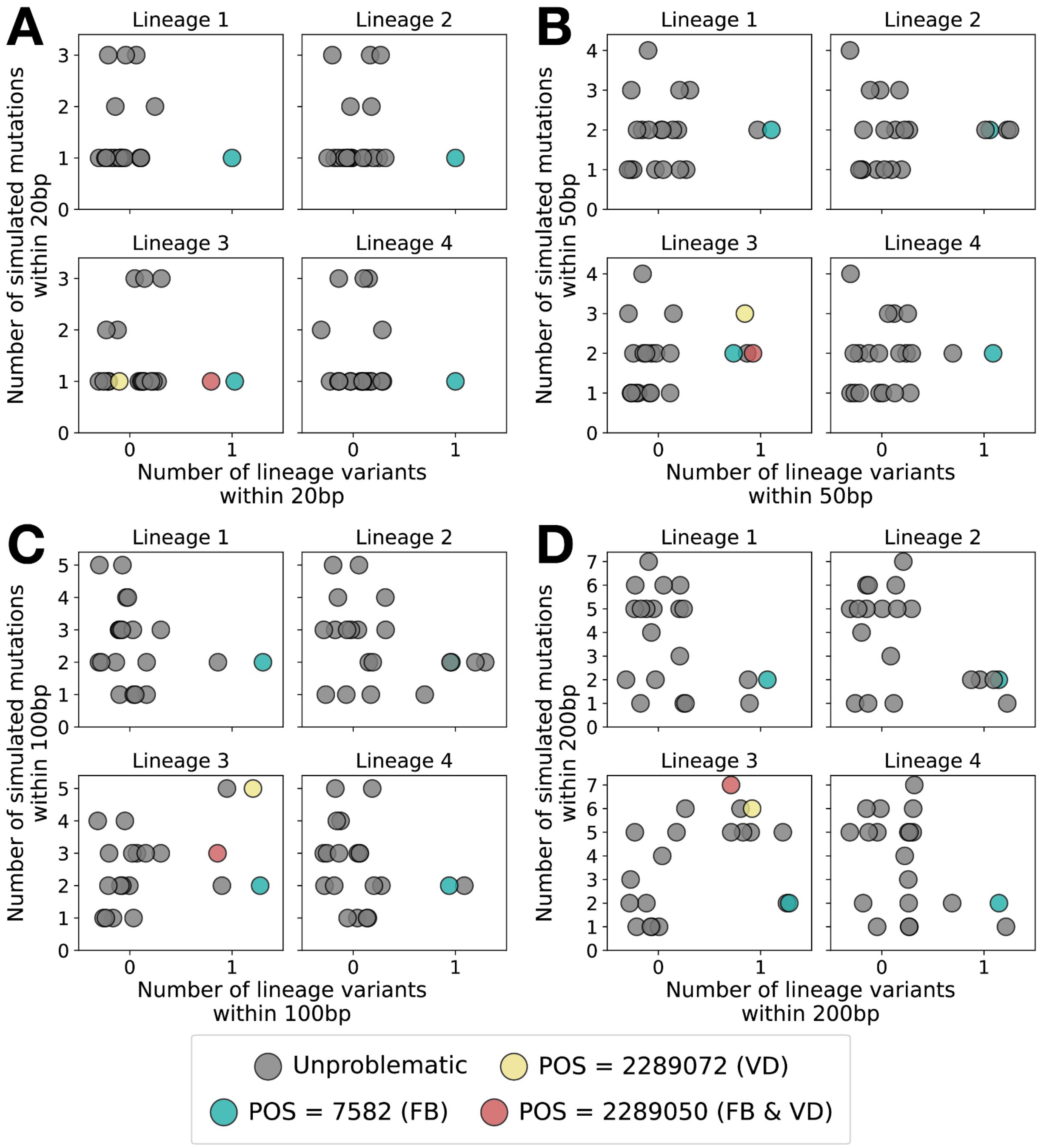

Figure S27 Number of nearby simulated mutations plotted against number of nearby lineage variants for each simulated DR mutation in the L1-4 strains for increasing window sizes. **a** Window size ±20bp. **b** Window size ±50bp. **c** Window size ±100bp. **d** Window size ±200bp. The three problematic mutations are colored as per the legend: POS = 7,582 was consistently missed only by FreeBayes in L1-4, POS = 2,289,050 was consistently missed by FreeBayes and VarDict in L3, and POS = 2,289,072 was consistently missed only by VarDict in L3.

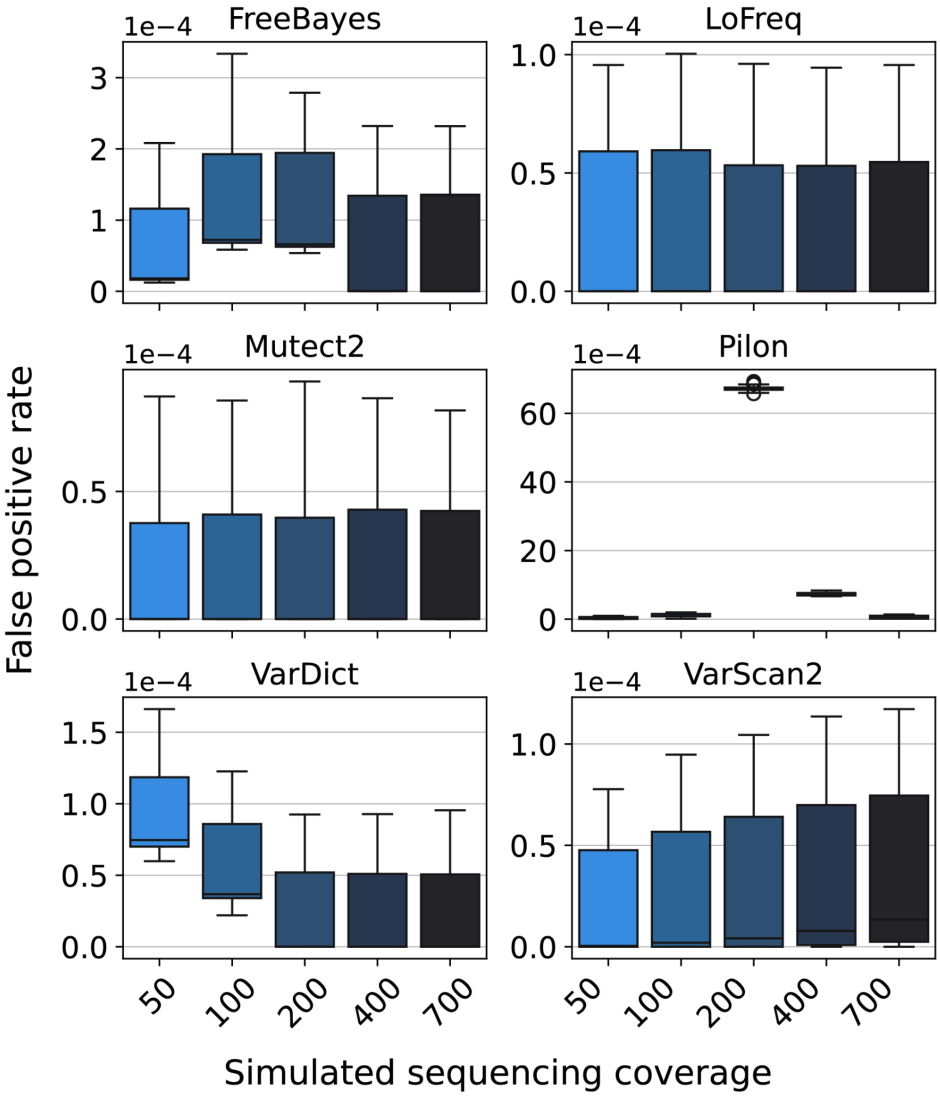

Figure S28 Genome-wide false positive rate across all strains in each ART-simulated sequencing coverage group. False positive rate is calculated per-base across the entire genome. The FPRs for FreeBayes in strains simulated at 100x-200x and for Pilon in strains simulated at 200x-400x is noticeably higher than the FPRs for those tools at other depths, which is consistent with the ISS-simulated data.

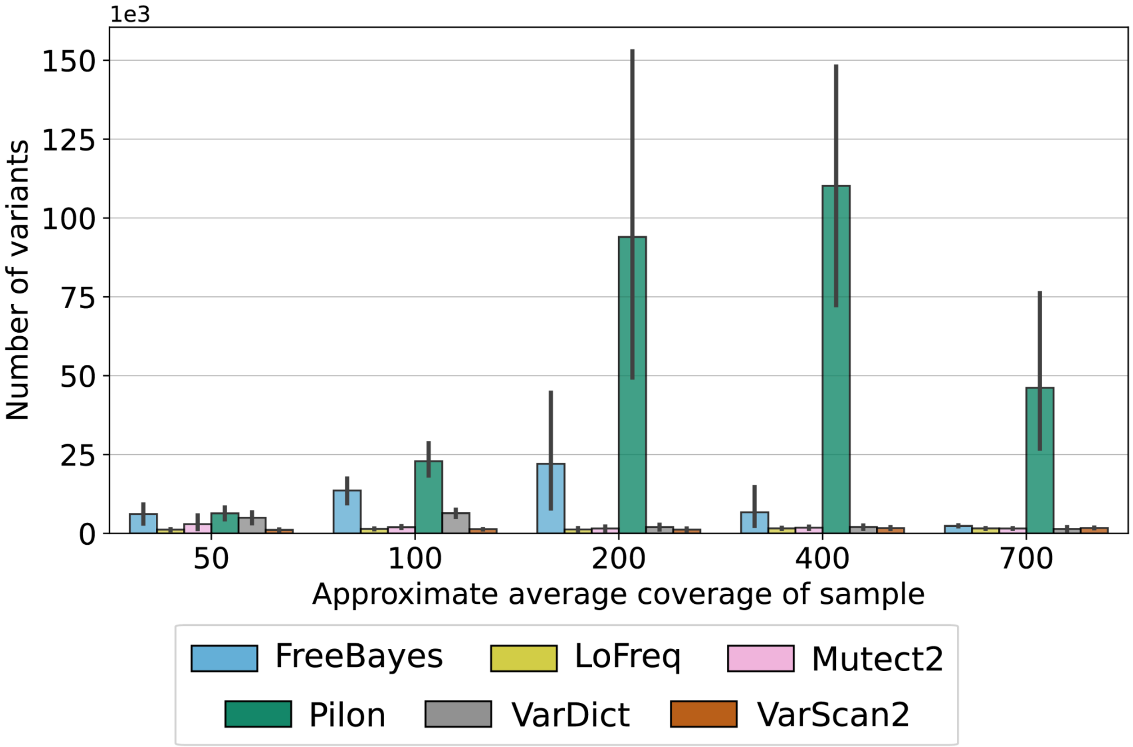

Figure S29 Distribution of total number of variants found by each tool in clinical isolates (n=20) with different average sequencing coverages. Each bar displays the average number of variants found per isolate by each tool, and the error bar indicates the 95% confidence interval.

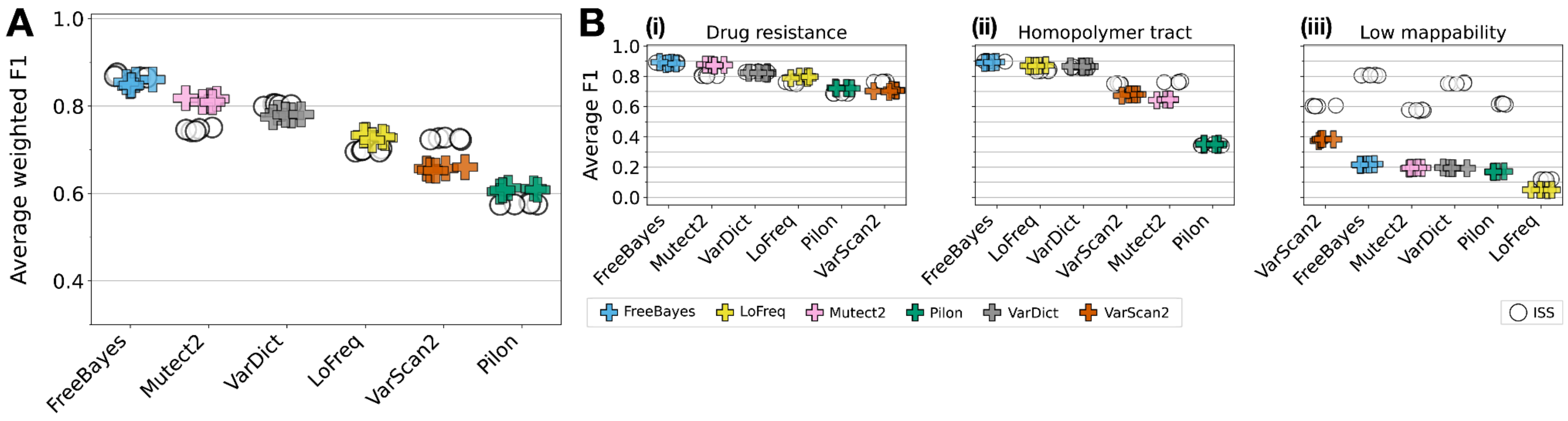

Figure S30 Overall variant caller accuracy in the ART-simulated data. **a** Weighted F1 score achieved by each variant caller in the H37Rv strains, averaged over simulated variant AFs, depths and mutation regions. Each point represents the average weighted F1 score for one of the replicate simulations. ISS data points are white. **b** Average F1 score achieved by each variant caller in the H37Rv strains (i) drug resistance, (ii) homopolymer tract, and (iii) low mappability regions. ISS data points are in white.

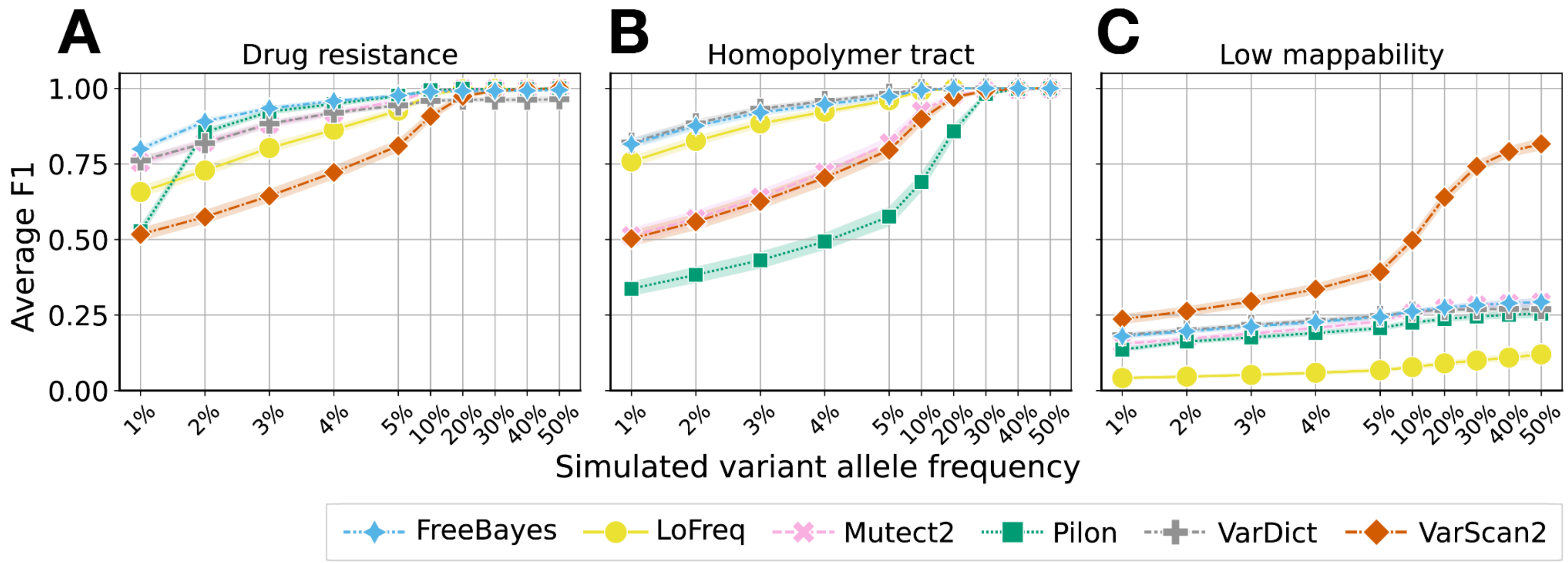

Figure S31 Average cumulative F1 across variant AF pooled over depths of 50x, 100x and 200x in the ART-simulated strains. **a** Average cumulative F1 in drug resistance regions (H37Rv and L1-4 strains). **b** Average cumulative F1 in homopolymer tract regions (H37Rv strains only). **c** Average cumulative F1 in low mappability regions (H37Rv strains only). To compute the average cumulative F1, we computed cumulative precision and recall as a function of increasing minimum variant AF for each of the six tools, averaged over haplotype, depths 50-200x and replicate. The band around each line represents the 95% confidence interval. Note that the x-axis tick gaps are not proportional to the actual simulated variant AF, and are larger for AF < 10% as this is where the greatest tool-wise differences occur.

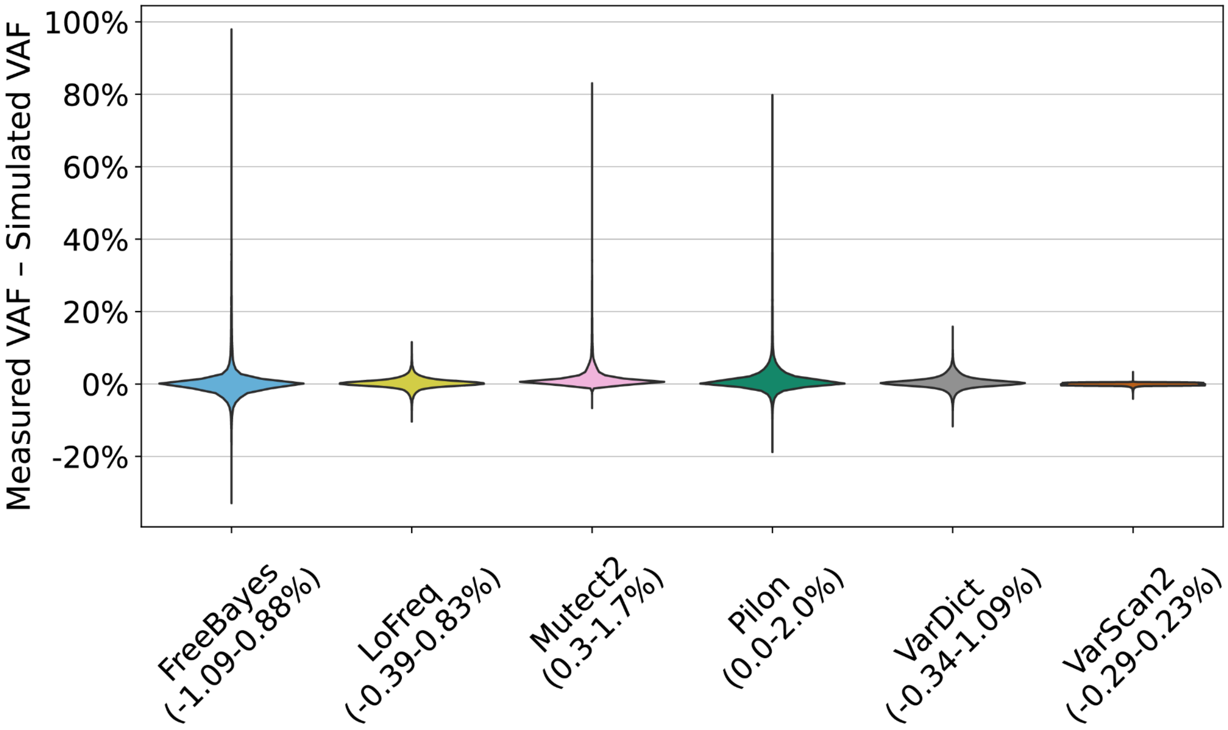

Figure S32 Distribution of the difference between the measured allele frequency and the simulated allele frequency for variants across all variants, depths, haplotypes and replicates (ART-simulated data).

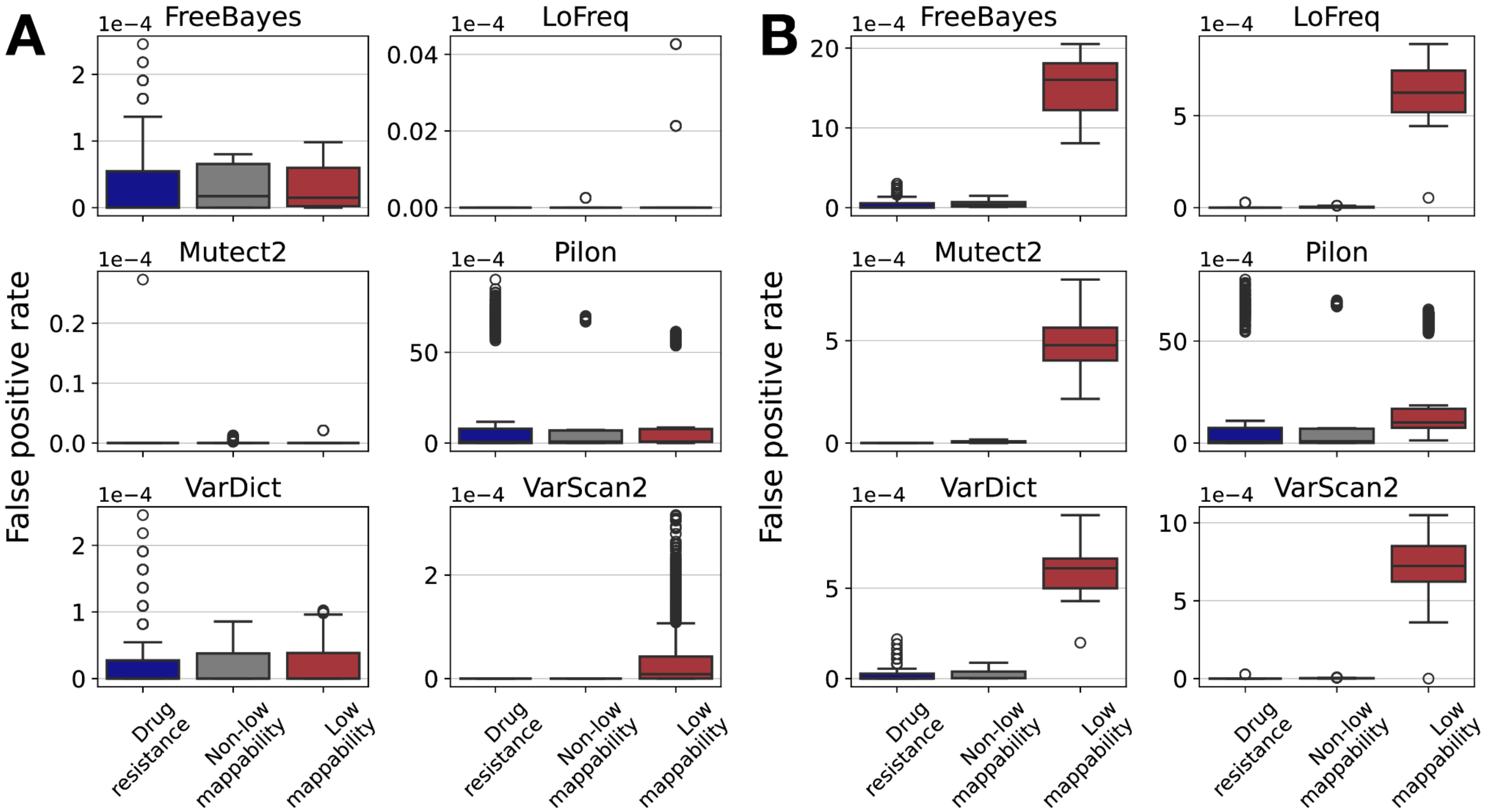

Figure S33 False positive rates by tool in drug resistance, non-low mappability and low mappability regions in the ART-simulated strains. **a** The distribution of false positive rates per strain and region in H37Rv strains. **b** The distribution of false positive rates per strain and region in L1-4 strains. Each region is defined to be mutually exclusive for this comparison i.e. the non-low mappability regions do not include the drug resistance regions. Each box plot shows the distribution of FPRs in each region and for each tool. Note that the subplots do not share the same y-axis. The pairwise comparisons between each region for each tool are statistically significant in both groups of strains after Benjamini-Hochberg correction (Mann-Whitney U test with FDR = 0.05).

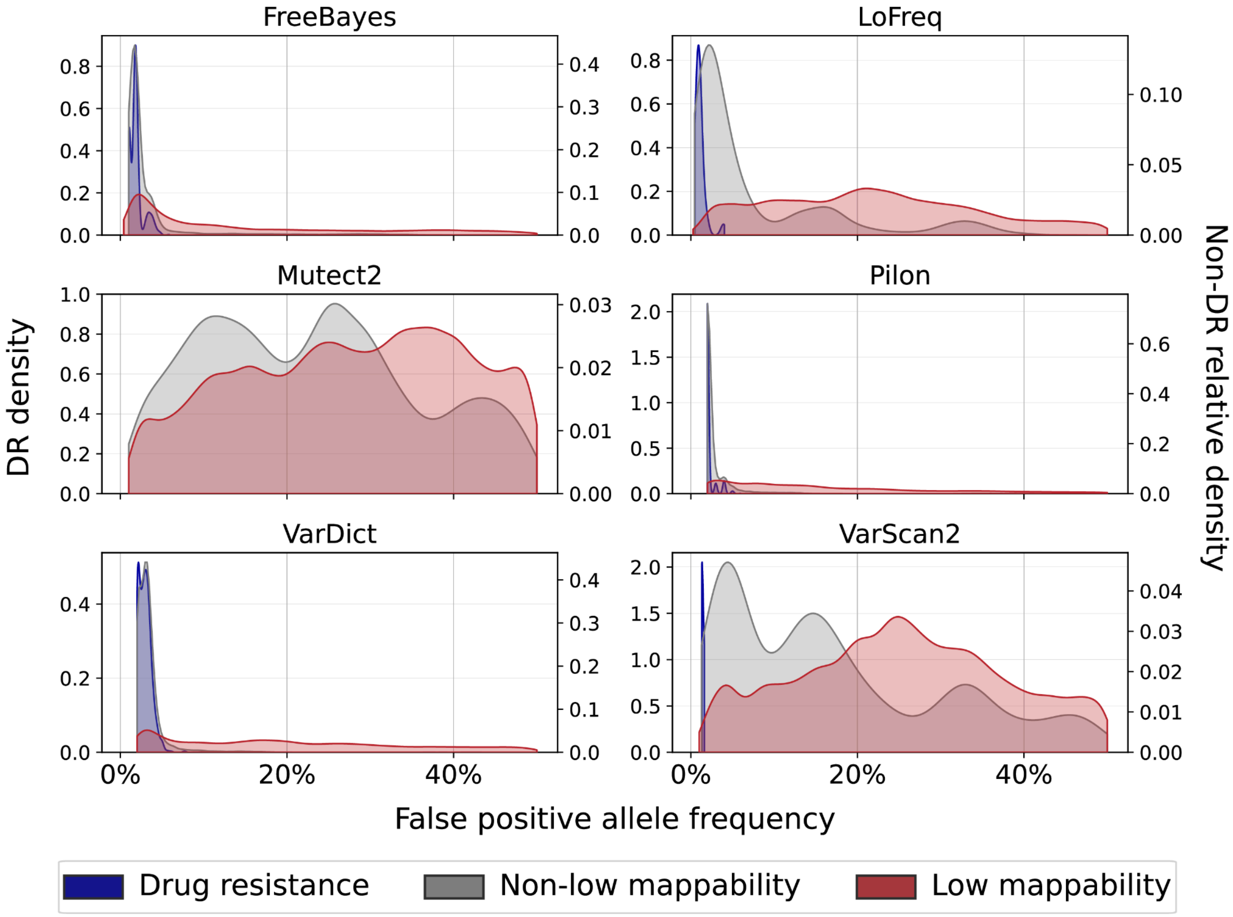

Figure S34 False positive allele frequency distribution in the ART-simulated L1-4 strains by regions. Each region is defined to be mutually exclusive for this comparison i.e. the non-low mappability regions do not include the drug resistance regions. The left y-axis displays the densities of the DR AF distributions, and the right y-axis displays the relative densities of the low mappability (non-DR) and non-low mappability (non-DR) AF distributions (normalized independently). For Pilon we include only the FP with AF > 1% (a median of 29% of Pilon FPs across all strains have AF = 1%). All DR FP occur at AF < 11% and FP in the other two regions occur at AFs 1-50%. Between 1-82% of the FP in non-low mappability regions occur at AF > 10%, while more than 60% of the LM FPs occur at AF > 10% for all tools except FreeBayes and Pilon (48% and 59% of the LM FPs have AF > 10% for these tools respectively).

Supplementary Tables

Table S1 Average DR-F1 per tool pooled across strains from an H37Rv and L1-4 background.

Tool	Average pooled F1	Average L1-4 F1 – average H37Rv F1
FreeBayes	0.86	-0.038*
VarDict	0.81	0.005
Mutect2	0.77	0.065*
VarScan2	0.73	-0.003
LoFreq	0.71	-0.005
Pilon	0.57	-0.003

The average difference in average DR F1 score (L1-4 – H37Rv) is displayed in the second column. The asterisks indicate the differences that were statistically significant after Benjamini-Hochberg correction (Mann-Whitney U test with FDR = 0.05).

Table S2 Difference in average DR F1 score for pairwise comparisons between each lineage (L1-4) for each tool.

Lineage 1	Lineage 2	FreeBayes	LoFreq	Mutect2	Pilon	VarDict	VarScan2
L1	L2	-0.014	-0.008	-0.008	-0.006	-0.013	-0.011
L1	L3	-0.024*	-0.009	0.004	0.001	-0.046*	-0.014
L1	L4	-0.004	-0.005	0.011	0.002	0.005	-0.013
L2	L3	-0.010*	-0.001	0.012	0.007	-0.033*	-0.003
L2	L4	0.010	0.003	0.019	0.008	0.018	-0.002
L3	L4	0.020*	0.004	0.007	0.001	0.051*	0.001

The value in each cell is the average DR F1 score achieved by a tool in “Lineage 1” subtracted from the average DR F1 score achieved by that tool in “Lineage 2,” for L1, L2, L3 and L4. The asterisks indicate the differences that were statistically significant after Benjamini-Hochberg correction (Mann-Whitney U test with FDR = 0.05). The only pairwise comparisons that were statistically significant were those involving L3 for FreeBayes and VarDict. We discuss the issue these tools had with consistently missed mutations in L3 in the Supplementary Results. The P-values for all other comparisons are >0.05.

Table S3 Average F1 score by tool and genomic region at depths 50-200x for increasing minimum variant allele frequency 1-50%.

Min. variant AF	Drug resistance						Homopolymer tract						Low mappability					
	FreeBayes	LoFreq	Mutect2	Pilon	VarDict	VarScan2	FreeBayes	LoFreq	Mutect2	Pilon	VarDict	VarScan2	FreeBayes	LoFreq	Mutect2	Pilon	VarDict	VarScan2
**1%**	0.80	0.61	0.72	0.51	0.78	0.59	0.83	0.71	0.63	0.33	0.82	0.58	0.70	0.09	0.46	0.63	0.70	0.44
**2%**	0.90	0.68	0.79	0.86	0.85	0.66	0.90	0.79	0.70	0.38	0.89	0.65	0.78	0.10	0.51	0.71	0.77	0.49
**3%**	0.94	0.76	0.85	0.93	0.91	0.73	0.94	0.85	0.77	0.43	0.94	0.72	0.83	0.11	0.56	0.78	0.83	0.54
**4%**	0.97	0.84	0.90	0.96	0.95	0.80	0.97	0.90	0.84	0.49	0.97	0.79	0.87	0.13	0.61	0.83	0.88	0.61
**5%**	0.98	0.91	0.94	0.98	0.96	0.87	0.98	0.95	0.90	0.57	0.99	0.87	0.91	0.15	0.67	0.88	0.92	0.69
**10%**	0.99	0.98	0.98	1.00	0.97	0.95	1.00	0.99	0.96	0.69	1.00	0.95	0.95	0.17	0.73	0.93	0.95	0.84
**20%**	0.99	1.00	0.99	1.00	0.97	0.99	1.00	1.00	0.98	0.86	1.00	0.99	0.96	0.21	0.80	0.95	0.95	0.94
**30%**	0.99	1.00	1.00	1.00	0.97	1.00	1.00	1.00	0.99	0.99	1.00	1.00	0.97	0.26	0.86	0.96	0.95	0.97
**40%**	0.99	1.00	1.00	1.00	0.97	1.00	1.00	1.00	0.99	1.00	1.00	1.00	0.97	0.33	0.89	0.96	0.96	0.97
**50%**	0.99	1.00	1.00	1.00	0.97	1.00	1.00	1.00	1.00	1.00	1.00	1.00	0.97	0.41	0.91	0.96	0.96	0.97

The value in each cell is the average F1 score for each tool in a specific genomic region, enforcing an increasing minimum variant allele frequency (AF) in each row. The F1 scores in drug resistance regions are averaged across strains from both an H37Rv and non-H37Rv background, while the F1 scores in the homopolymer tract and low mappability regions are for strains from an H37Rv background only.

Table S4 Median FPR across H37Rv and L1-4 strains per tool and overall by region.

	FreeBayes	LoFreq	Mutect2	Pilon	VarDict	VarScan2	Overall
**DR**	0.00	0.00	0.00	1.09E-04	0.00	0.00	0.00
**Non-LM**	3.23E-05	0.00	0.00	1.11E-04	2.56E-06	0.00	1.54E-06
**LM**	6.40E-05	0.00	0.00	9.40E-04	2.77E-05	2.35E-05	4.10E-06

This table describes the false positive rate (FPR) in drug resistance, non-low mappability and low mappability regions. Each region is defined to be mutually exclusive for this comparison i.e. the non-low mappability regions do not include the drug resistance regions. LoFreq and Mutect achieve the lowest median FPR across all strains. The median FPR is lowest in drug resistance resistance and highest in low mappability regions for all tools.

Table S5 Percentage of FP detected in LM regions and at AF = 1% by each tool for the H37Rv simulations and L1-4 simulations separately.

Tool	Median number of FP		Median % LM FP		Median % FP at AF = 1%	
	**H37Rv**	**L1-4**	**H37Rv**	**L1-4**	**H37Rv**	**L1-4**
Pilon	481	968	10.91%	53.14%	99.96%	41.72%
FreeBayes	141	894	13.54%	79.64%	0.00%	0.00%
VarDict	130	410	10.00%	95.26%	0.00%	0.00%
VarScan2	13	428	100.00%	97.40%	0.00%	0.00%
LoFreq	1	370	0.00%	95.81%	0.00%	0.00%
Mutect2	2	311	0.00%	76.13%	0.00%	0.00%

For each tool we list the median total number of genome-wide FP detected at all variant AFs in H37Rv and L1-4 separately (first two columns), the percentage of these total FP that occur in low mappability regions (second two columns), and the percentage of these FP total FP that occur at AF = 1%. The tools are sorted in descending order by the median total number of FP detected (H37Rv + L1-4).

Table S6 SNV filtering of false and true variants called by FreeBayes (AF < 5%) in L1-4 strains.

Sequencing coverage	Total		Percentage filtered					
	Pre-filtering		F1: Error model filtering		F2: Error model + hard filtering		F3: Error model + hard filtering + region masking	
	FP	TP	FP	TP	FP	TP	FP	TP
50x	18618	337	30.0%	0.4%	100.0%	100.0%	100.0%	100.0%
100x	60338	815	23.3%	0.1%	99.6%	85.8%	100.0%	86.9%
200x	42259	1049	43.1%	0.4%	97.8%	45.0%	99.7%	47.4%
400x	28625	1212	65.8%	0.0%	94.7%	8.8%	99.2%	14.4%
700x	29253	1261	65.3%	0.0%	92.2%	0.9%	99.0%	6.4%
Overall	179093	4674	45.5%	0.7%	96.9%	46.8%	99.6%	49.8%

The pre-filtering group of columns displays the total number of FP and TP SNVs called by FreeBayes across all strains simulated at a specific sequencing depth. A total of 80 strains are considered in each sequencing depth group (four variant AFs 1-4%, four background genomes, five replicates). The F1-F3 groups of columns correspond to each of the three sequential SNV filtering schemes and display the average percentages of FPs and TPs filtered out per strain. F1 (Filter 1): error model filtering, F2: error model filtering and hard filtering (forward and reverse strand allele counts ≥ 2, depth ≥ 5, mapping quality ≥ 40), F3: error model filtering, hard filtering and region masking (low mappability regions and rRNA genes). FP and TP statistics are broken down by simulated sequencing coverage group, and summarized overall. Note for F3 that we simulated variants in the regions masked in this filtering scheme.

Table S7 INDEL filtering of false and true variants called by FreeBayes (AF 5-50%) in L1-4 and H37Rv strains respectively.

Sequencing coverage	Total		Percentage filtered					
	Pre-filtering		F1: AF adjustment		F2: AF adjustment + hard filtering		F3: AF adjustment + hard filtering + region masking	
	FP	TP	FP	TP	FP	TP	FP	TP
50x	2527	2616	45.4%	1.6%	61.1%	23.9%	98.8%	23.9%
100x	2698	2624	45.1%	2.1%	57.6%	6.7%	98.8%	6.7%
200x	2754	2636	43.1%	1.0%	53.8%	1.4%	98.9%	1.4%
400x	2800	2603	41.5%	0.4%	52.0%	0.4%	98.9%	0.4%
700x	2802	2582	39.9%	0.0%	49.3%	0.0%	99.0%	0.0%
Overall	13581	13061	43.0%	1.0%	54.8%	6.5%	98.9%	6.5%

The pre-filtering group of columns displays the total number of FP and TP INDELs called by FreeBayes across all strains simulated at a specific sequencing depth. A total of 120 L1-4 strains are considered in each sequencing depth group for the FP statistics (six variant AFs 5-50%, four background genomes, five replicates), and a total of 300 H37Rv strains are considered in each sequencing depth group for the TP statistics (six variant AFs 5-50%, ten haplotypes, five replicates). The F1-F3 groups of columns correspond to each of the three sequential INDEL filtering schemes and display the average percentages of FPs and TPs filtered out per strain. F1 (Filter 1): filtering after AF adjustment, F2: filtering after AF adjustment and hard filtering, F3: filtering after AF adjustment, hard filtering and region masking (low mappability regions, rRNA genes and sites within 100bp of an insertion sequence or phage). FP and TP statistics are broken down by simulated sequencing coverage group, and summarized overall. Note for F3 that we simulated variants in the regions masked in this filtering scheme but these variants were not detected by FreeBayes in most strains in the first place.

Table S8 Median number of low-frequency variants found in DR, HT, LM and all other regions.

Genomic region	Median number of low-frequency variants
Drug resistance	0
Homopolymer tract	1
Low mappability	4
Other	106

The number of low-frequency variants per strain did not differ by (1) lineage, or (2) average sequencing coverage after multiple Mann-Whitney U tests (Benjamini-Hochberg correct, FDR = 0.05).

Table S9 Average difference between the simulated AF and the expected AF in each region for ISS and ART.

	ISS			ART		
	**DR**	**HT**	**LM**	**DR**	**HT**	**LM**
**AF < 10%**	-0.012%	-0.352%	-0.963%	0.006%	-0.301%	-1.825%
**AF ≥ 10%**	-0.652%	-3.443%	-10.285%	-0.413%	-3.170%	-18.237%

This table describes the average difference between the simulated AF and the expected AF in drug resistance (DR), homopolymer tract (HT) and low mappability (LM) regions. The average difference for DR regions includes both H37Rv and L1-4 strains. The largest average differences between the simulated and expected AFs occur in LM regions.
